# TXNIP Deficiency Exacerbates Endotoxic Shock via the Induction of Excessive Nitric Oxide Synthesis

**DOI:** 10.1371/journal.ppat.1003646

**Published:** 2013-10-03

**Authors:** Young-Jun Park, Sung-Jin Yoon, Hyun-Woo Suh, Dong Oh Kim, Jeong-Ran Park, Haiyoung Jung, Tae-Don Kim, Suk Ran Yoon, Jeong-Ki Min, Hee-Jun Na, Seon-Jin Lee, Hee Gu Lee, Young Ho Lee, Hee-Bong Lee, Inpyo Choi

**Affiliations:** 1 Immunotherapy Research Center, Korea Research Institute of Bioscience and Biotechnology, Yuseong-gu, Daejeon, Republic of Korea; 2 Department of Functional Genomics, University of Science and Technology, Yuseong-gu, Daejeon, Republic of Korea; 3 Department of Biomolecular Science, University of Science & Technology, Yuseong-gu, Daejeon, Republic of Korea; 4 Regenerative Medicine Research Center, Korea Research Institute of Bioscience and Biotechnology, Yuseong-gu, Daejeon, Republic of Korea; 5 Medical Genomics Research Center, Korea Research Institute of Bioscience and Biotechnology, Yuseong-gu, Daejeon, Republic of Korea; 6 Department of Anatomy, School of Medicine, Chungnam National University, Chung-gu, Daejeon, Republic of Korea; 7 Department of Biochemistry, College of Natural Sciences, Kangwon National University, Chuncheon, Republic of Korea; Harvard Medical School, United States of America

## Abstract

Thioredoxin-interacting protein (TXNIP) has multiple functions, including tumor suppression and involvement in cell proliferation and apoptosis. However, its role in the inflammatory process remains unclear. In this report, we demonstrate that *Txnip^−/−^* mice are significantly more susceptible to lipopolysaccharide (LPS)-induced endotoxic shock. In response to LPS, *Txnip^−/−^* macrophages produced significantly higher levels of nitric oxide (NO) and inducible nitric oxide synthase (iNOS), and an iNOS inhibitor rescued *Txnip^−/−^* mice from endotoxic shock-induced death, demonstrating that NO is a major factor in TXNIP-mediated endotoxic shock. This susceptibility phenotype of *Txnip^−/−^* mice occurred despite reduced IL-1β secretion due to increased *S*-nitrosylation of NLRP3 compared to wild-type controls. Taken together, these data demonstrate that TXNIP is a novel molecule that links NO synthesis and NLRP3 inflammasome activation during endotoxic shock.

## Author Summary

TXNIP has many biological functions, including the inhibition of tumor growth, suppression of hepatocarcinogenesis, and regulation of glucose metabolism and reactive oxygen species (ROS) generation in different cell types. However, little is known about its role in the inflammatory process. In this study, our results demonstrate that TXNIP plays a critical role in the control of lethal endotoxin-induced shock by controlling NO production in innate immune cells via the regulation of iNOS expression. This regulation is mediated through changes in the activation and translocation of NF-κB that affect the NF-κB/iNOS pathway. In addition, excessive NO reduces the production of IL-1β via *S*-nitrosylation of the NLRP3 inflammasome. Subsequently, the survival of *Txnip^−/−^* mice is significantly decreased due to hypothermia and hypoglycemia. Overall, these results suggest that TXNIP is a novel therapeutic target for the treatment of inflammatory diseases.

## Introduction

Thioredoxin-interacting protein (TXNIP) was originally described as a differentially expressed gene in 1α,25-dihydroxyvitamin D3 (1,25[OH]2D3)-treated HL-60 cells [1] and as a negative regulator of the function and expression of thioredoxin [2]. TXNIP is a member of the arrestin protein family, which contains 2 characteristic arrestin-like domains: a PXXP sequence, which is a known binding motif for SH3 domain-containing proteins, and a PPXY sequence, which is a known binding motif for the WW domain [3]. TXNIP interacts with thioredoxin, inhibiting its antioxidant activity, and the protein is believed to be involved in a wide variety of cellular processes, including the response to oxidative stress [2], [4], cell proliferation, and apoptosis [5]. In addition, the strong reduction in TXNIP protein expression in various types of tumors suggests that this protein functions as a tumor suppressor. Furthermore, overexpression of TXNIP in melanoma cells results in the inhibition of metastasis, suggesting that TXNIP can suppress metastasis [6]–[8]. Although TXNIP is also highly expressed in immune cells, the specific regulatory effects of TXNIP on the inflammatory process have only recently been examined.

Nitric oxide (NO) is one of important cellular signaling molecules involved in many physiological processes as well as the killing of intracellular pathogens [9]. The production of NO is regulated by a family of nitric oxide synthases (NOSs), and different NOS subtypes are expressed depending on the tissue type [10]. Mice deficient in iNOS genes are more resistant to lipopolysaccharide (LPS)-induced acute lung injury than wild-type mice [11]. Bioactive NO is physiologically generated through several different mechanisms, including enzymatic reduction by components of the mitochondrial respiratory chain, xanthine oxidase, endothelial NO synthase (eNOS), and cytochrome P450, as well as nonenzymatic mechanisms such as acidic disproportionation [12].

It has been shown that TXNIP has a relationship with the NLRP3 inflammasome [13], [14]. The NLRP3 inflammasome is a multiprotein complex that mediates the activation of caspase-1, which in turn mediates the processing of important mediators of innate immunity such as IL-1β [15], [16]. It was recently reported that NO contributes to suppressing the production of IL-1β by *S*-nitrosylating the NLRP3 inflammasome [17], [18]. However, little is known about the regulatory mechanism for NO-mediated NLRP3 inflammasome inhibition and the potential role for TXNIP in this progression.

In this study, we found that *Txnip^−/−^* mice were extremely susceptible to LPS-induced endotoxic shock as a result of increased production of NO and iNOS. These results indicated that TXNIP is a crucial constituent of the inflammatory response through the regulation of NO production via the NF-κB/iNOS pathway. In addition, we also observed increased NO production in *Txnip^−/−^* cells, which suppressed NLRP3 inflammasome activation, indicating a critical role for TXNIP in the activation of the NLRP3 inflammasome during endotoxic shock.

## Results

### *Txnip^−/−^* mice are extremely susceptible to LPS-induced endotoxic shock

**Figure 1:**
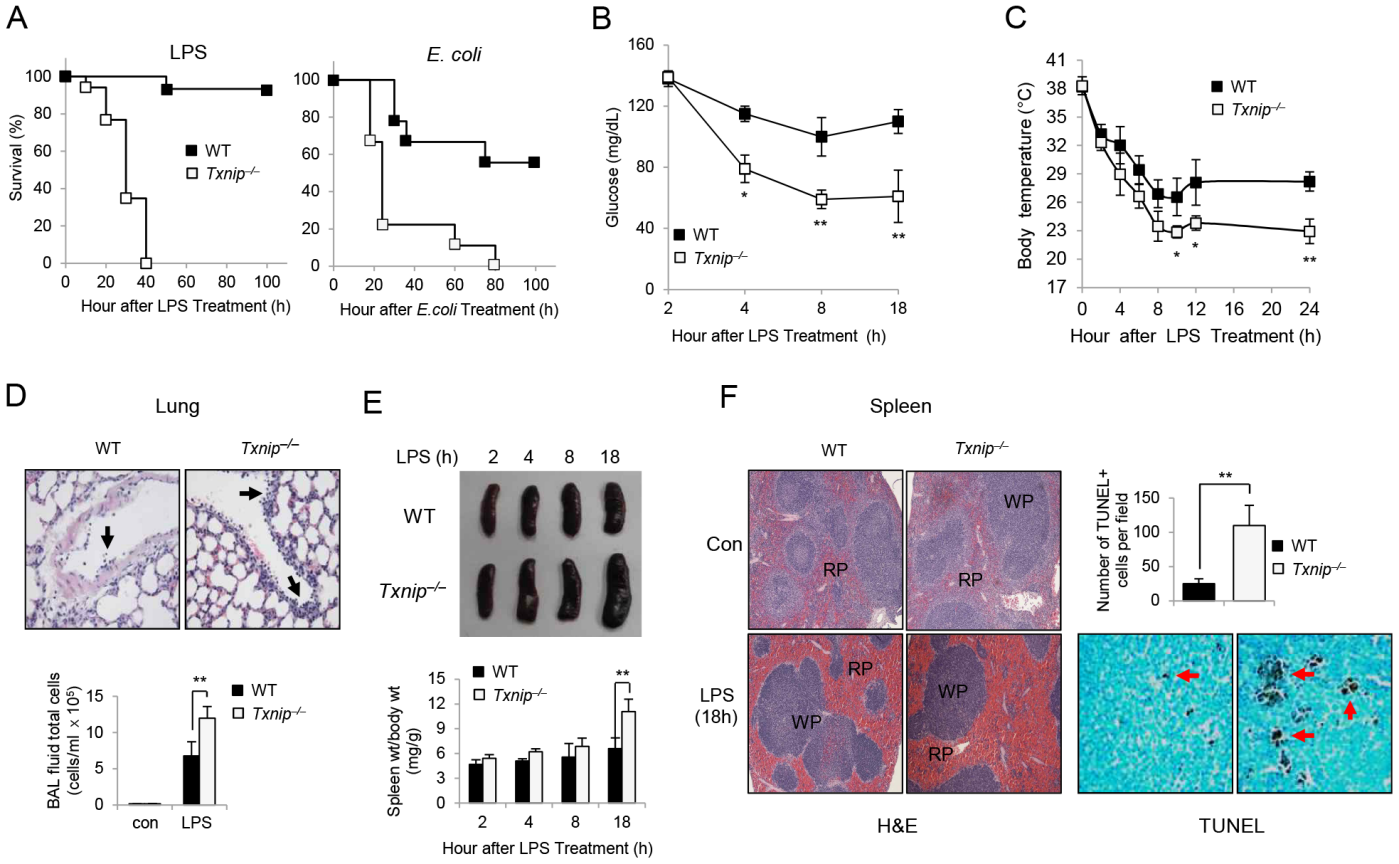
*Txnip^−/−^* mice are hypersusceptible to endotoxic shock as compared to WT mice. (A) Left panel, LPS (10 mg/kg body weight) was injected i.p. into WT mice (n = 17) and *Txnip^−/−^* mice (n = 17). Right panel, *E. coli* (10^8^ CFU of live *E. coli* (DH5α)) was injected i.p. into WT mice (n = 9) and *Txnip^−/−^* mice (n = 9). (B and C) After LPS injection, the blood glucose and body temperature were measured in WT and *Txnip^−/−^* mice at the indicated times. Values represent the mean ± SD from 4 mice at each time point. Significant differences between WT and *Txnip^−/−^* mice at the indicated time points are denoted with *P<0.05 and **P<0.01. Data are representative of 3 independent experiments. (D) Enhanced cell infiltration in the lungs of LPS-challenged TXNIP^−/−^ mice (right). BAL fluid was collected 18 h after LPS administration from WT or *Txnip^−/−^* mice, and total cells counts in the BAL fluid were determined by optical microscopy after cytocentrifugation and H&E staining. Original magnification (400×). The arrows indicate immune cells in the blood vessel. (E) Size of the spleens from WT and *Txnip^−/−^* mice at 2, 4, 8, and 18 h after injection of LPS. Quantitation of the spleen size is shown. (F) Spleen histology with H&E staining, original magnification (100×). TUNEL assays were performed on sections of spleens from mice at 18 h post-LPS injection. The arrows denote apoptotic cells (WP, white pulp; RP, red pulp). Quantitation of the TUNEL staining is shown. We counted apoptotic cells within 6 randomly selected fields. The data shown in (A) are presented as Kaplan-Meyer curves from 3 independent experiments. The data shown in D-F are representative of 3 independent experiments with 3 mice at each time point (**P<0.01).

TXNIP is a stress-induced protein, although the function of TXNIP in LPS-induced endotoxic shock is unclear. Therefore, we sought to determine whether TXNIP deficiency would affect the susceptibility to endotoxic shock *in vivo*. Following the injection of LPS (10 mg/kg body weight), the survival of mice was assessed every 10 h. Almost all of the wild-type (WT, n = 17) mice survived, whereas all of the *Txnip^−/−^* mice died within 40 h (*Txnip^−/−^*, n = 17) of LPS injection (Fig. 1 A, left). Likewise, WT and *Txnip^−/−^* mice injected with *E. coli* also displayed similar mortality patterns (Fig. 1 A, right). Overall, the significantly decreased survival of *Txnip^−/−^* mice suggests that TXNIP plays a vital role in regulating the sensitivity of mice to lethal endotoxin-induced shock *in vivo*. In agreement with these data, the blood glucose level and body temperature of the *Txnip^−/−^* mice were lower than those of WT mice (Fig. 1, B and C). The blood glucose level in *Txnip^−/−^* mice decreased faster, and the body temperature of *Txnip^−/−^* mice at 24 h post-injection was approximately 5°C lower than the body temperature of WT mice.

Eighteen hours after LPS injection, we observed that the lungs from LPS-treated *Txnip^−/−^* mice showed more intense immune cell infiltration in comparison to WT lungs (Fig. 1 D). In addition, the spleens from LPS-treated *Txnip^−/−^* mice were significantly larger than those of WT mice (Fig. 1 E). Furthermore, apoptosis was analyzed in splenocytes using hematoxylin and eosin (H&E) staining and TUNEL (terminal deoxynucleotidyl transferase dUTP nick-end labeling) assays 18 h after LPS injection, and a large number of apoptotic cells were detected in the white pulp of the spleens from the *Txnip^−/−^* mice (Fig. 1 F). Overall, these data suggest that TXNIP plays an essential role in regulating sensitivity to lethal endotoxin-induced shock.

### Deletion of TXNIP does not affect the production of inflammatory cytokines

**Figure 2:**
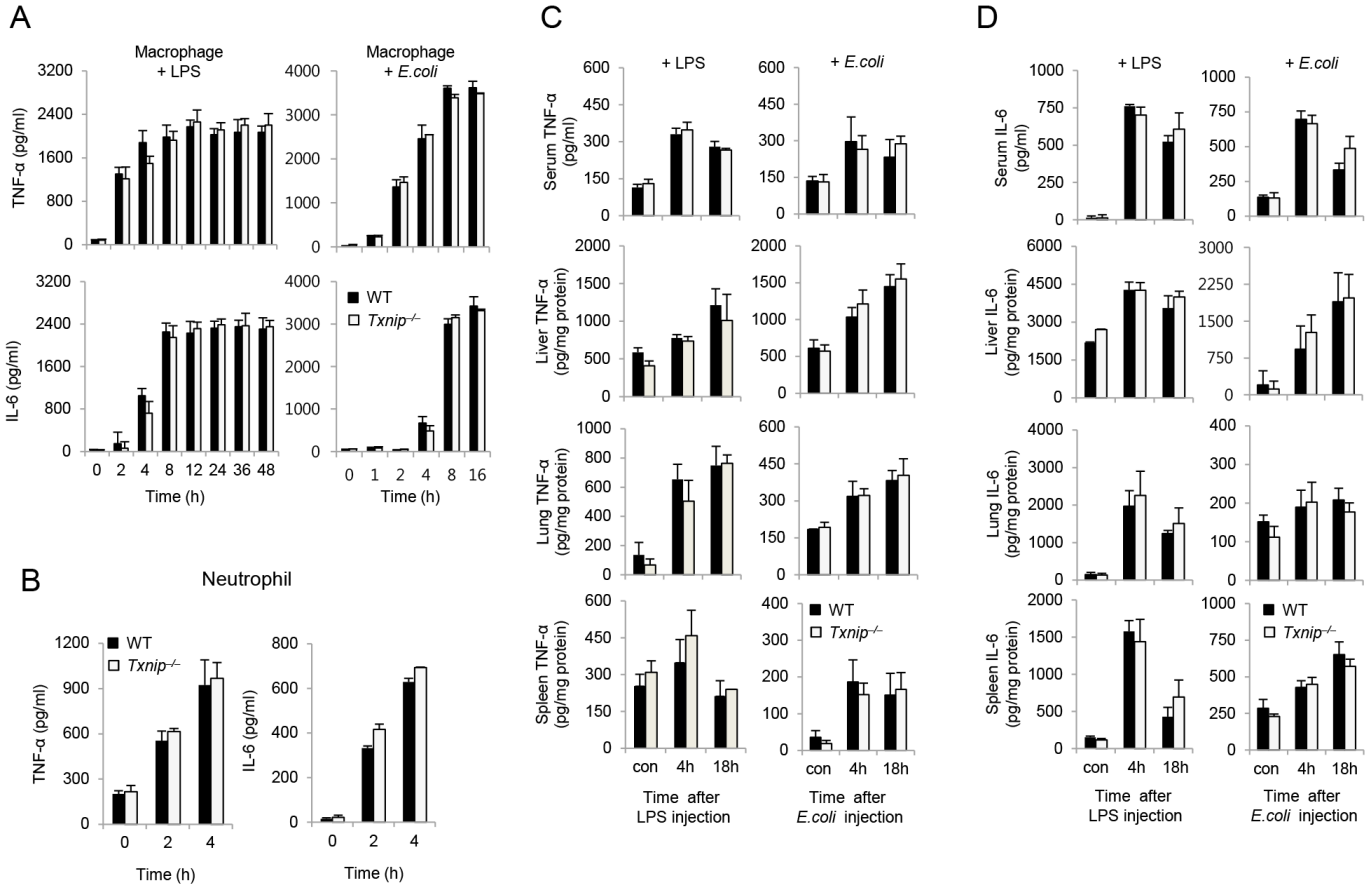
TXNIP does not affect the production of inflammatory cytokines by macrophages or neutrophils. (A) Peritoneal macrophages from WT or *Txnip^−/−^* mice were treated with 100 ng/ml LPS or *E. coli* at a multiplicity of infection (MOI) of 10. The data shown are representative of 3 independent experiments with 3 mice at each time point. (B) BM neutrophils from WT or *Txnip^−/−^* mice were treated with 100 ng/ml LPS, and then their supernatants were harvested at the indicated times. TNF-α and IL-6 were measured in the culture supernatants by ELISA. A representative experiment using neutrophils pooled from 2 WT or *Txnip^−/−^* mice and performed in triplicate is shown. Three additional experiments with separate groups of mice demonstrated similar results. WT mice (n = 5 per group) and *Txnip^−/−^* mice (n = 5 per group) were injected with LPS (10 mg/kg body weight) or *E. coli* (10^8^ CFU). Following these injections, TNF-α (C) and IL-6 (D) were measured in the sera or tissue lysates (liver, lung, and spleen) from WT and *Txnip^−/−^* mice at the indicated time points. The data shown in (C) and (D) are representative of 3 independent experiments with 3 mice at each time point.

It is well-known that proinflammatory cytokines are one of main mediators of lethal endotoxin-induced shock [19]. Therefore, we examined the production of inflammatory cytokines, including TNF-α and IL-6, during LPS- or *E. coli*-induced inflammatory responses. First, we analyzed the production of LPS- or *E. coli*-induced cytokines by peritoneal macrophages and LPS-induced cytokines by bone marrow (BM) neutrophils, as both of these cell types are the main producers of proinflammatory cytokines in response to LPS or *E. coli*. Surprisingly, the production TNF-α and IL-6 by peritoneal macrophages from *Txnip^−/−^* mice in response to LPS or *E. coli* was not significantly increased (Fig. 2 A). Similarly, WT and *Txnip^−/−^* neutrophils treated with 100 ng/ml LPS also produced similar levels of TNF-α and IL-6 (Fig. 2 B).

To confirm these levels of cytokine production *in vivo*, we analyzed the level of TNF-α (Fig. 2 C) and IL-6 (Fig. 2 D) in the serum, liver, lungs, and spleen of WT and *Txnip^−/−^* mice following treatment with LPS or *E. coli*. At both 4 h and 18 h after LPS or *E. coli* treatment, the concentration of cytokines in the liver, spleen, and serum was increased to similar levels in both groups of mice. Collectively, these data suggest that the increased sensitivity of *Txnip^−/−^* mice to endotoxin-induced shock is not the result of increased production of proinflammatory cytokines in response to LPS.

### The expression of iNOS and the production of NO are significantly increased in *Txnip^−/−^* mice in response to LPS

**Figure 3:**
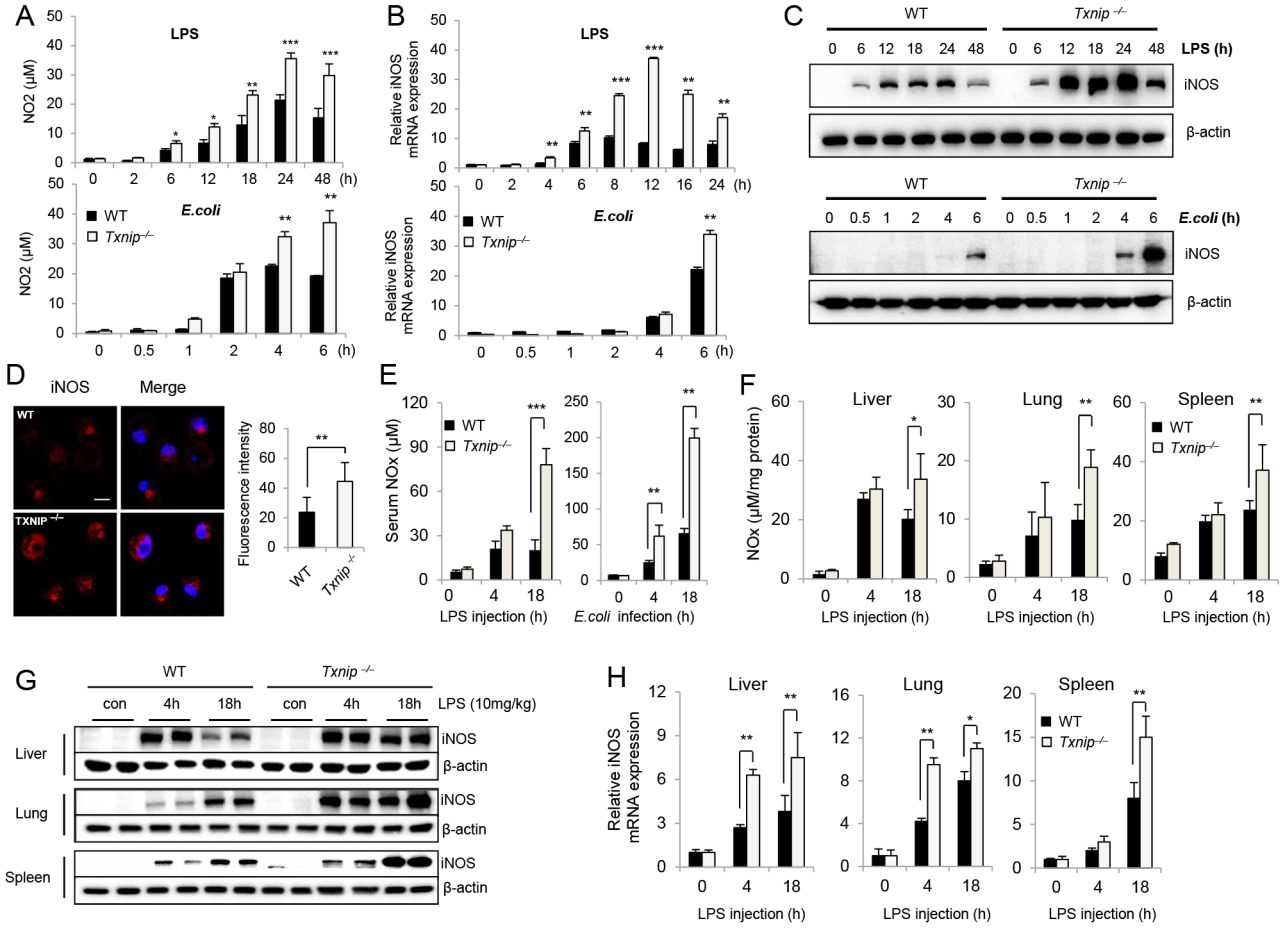
The expression of iNOS and the production of NO are significantly elevated in LPS-treated *Txnip^−/−^* mice. Peritoneal Macrophages from WT and *Txnip^−/−^* mice on the C57BL/6 background were treated with 100 ng/ml LPS or *E. coli* at a MOI of 10, and cell lysates and supernatants were harvested at the indicated times. (A) NO production in response to LPS or *E. coli* was determined by measuring the amount of nitrite in the culture media using Griess reagents. (B) iNOS mRNA expression levels were assessed by real-time PCR, and (C) iNOS protein levels were determined by immunoblotting with an anti-iNOS antibody. One representative experiment using macrophages pooled from 3 WT and *Txnip^−/−^* is shown. Three additional experiments with separate groups of mice provided similar results (data shown in A-C). (D) Expression levels of iNOS in macrophages from WT and *Txnip^−/−^* mice were determined by performing immunofluorescence with an antibody specific for iNOS (conjugated to the red fluorescent dye Alexa Fluor 546 (Alexa 546)). Then, LPS-treated cells were fixed, permeabilized, and analyzed by confocal microscopy (Carl Zeiss, LSM510). Confocal images were processed using the program Metamorph 6.1 (Universal Imaging, Media, PA). The graph represents 3 independent experiments in which 10 cells were analyzed for each condition. Confocal images were processed using the program Metamorph 6.1 (Universal Imaging, Media, PA). **P<0.01 versus WT. Scale bar, 20 µm. (E) After LPS or *E. coli* injection in WT (n = 5 per group) and *Txnip^−/−^* mice (n = 5 per group), serum NO levels were determined. (**P<0.01, ***P<0.001). (F) Liver, lung, and spleen samples were obtained from WT (n = 5 per group) and *Txnip^−/−^* mice (n = 5 per group) at 0, 4, and 18 h after LPS administration. Tissue lysates were prepared by homogenization. Nitrite plus nitrate (NOx) was measured in the serum, and tissue lysates were measured using a nitrate reductase-based colorimetric assay kit. These data are representative of at least 3 independent experiments (*P<0.05, **P<0.01, ***P<0.001). (G) Tissue lysate samples from (F) were analyzed by SDS-PAGE and immunoblotting with an anti-iNOS antibody. (H) Real-time PCR analysis of iNOS mRNA expression in the liver, lung, and spleen from WT and *Txnip^−/−^* mice. Both types of mice were injected with LPS (10 mg/kg) for the indicated time periods. Data are presented as the mean ± SD of 3 independent experiments (*P<0.05; **P<0.01).

NO is another inflammatory mediator involved in lethal endotoxin-induced shock [9]. To examine the possible role of NO in the increased sensitivity of *Txnip^−/−^* mice to LPS or *E. coli*, we analyzed the production of NO by murine peritoneal macrophages from WT and *Txnip^−/−^* mice. Interestingly, NO production was significantly increased in *Txnip^−/−^* macrophages after LPS or *E. coli* stimulation as compared to WT macrophages (Fig. 3 A). In addition, peritoneal macrophages from *Txnip^−/−^* mice showed increased iNOS mRNA and protein expression levels (Fig. 3, B and C). Furthermore, using confocal microscopy, we found that large amounts of iNOS were induced in *Txnip^−/−^* macrophages 18 h after LPS stimulation (Fig. 3 D). Conversely, the expression of iNOS in WT macrophages was decreased in comparison to *Txnip^−/−^* macrophages.

Next, LPS- or *E. coli*-induced NO production in the serum of WT and *Txnip^−/−^* mice was measured *in vivo*. While NO was generally similar between WT and *Txnip^−/−^* mice 4 h after injection, it was significantly elevated in the serum (Fig. 3E) and organs (Fig. 3F) of the *Txnip^−/−^* mice 18 h after injection. These results suggest that NO may have a crucial role in the increased sensitivity of *Txnip^−/−^* mice to LPS. We also measured the expression of iNOS in the liver, lungs, and spleen after LPS injection, and similar to the results observed for NO production, iNOS expression was markedly increased in *Txnip^−/−^* mice compared to WT mice (Fig. 3 G). In addition, each organ from *Txnip^−/−^* mice displayed increased iNOS mRNA levels (Fig. 3 H). As Forrester *et al.* previously reported that the half-life of the TXNIP protein is very short [20], we analyzed the expression of TXNIP in mice following the administration of LPS. TXNIP expression was significantly decreased within 2 h of LPS injection but had recovered completely after 16 h (Fig. S1).

### Inhibition of iNOS prevents the increased NO levels in *Txnip^−/−^* mice after LPS injection

**Figure 4:**
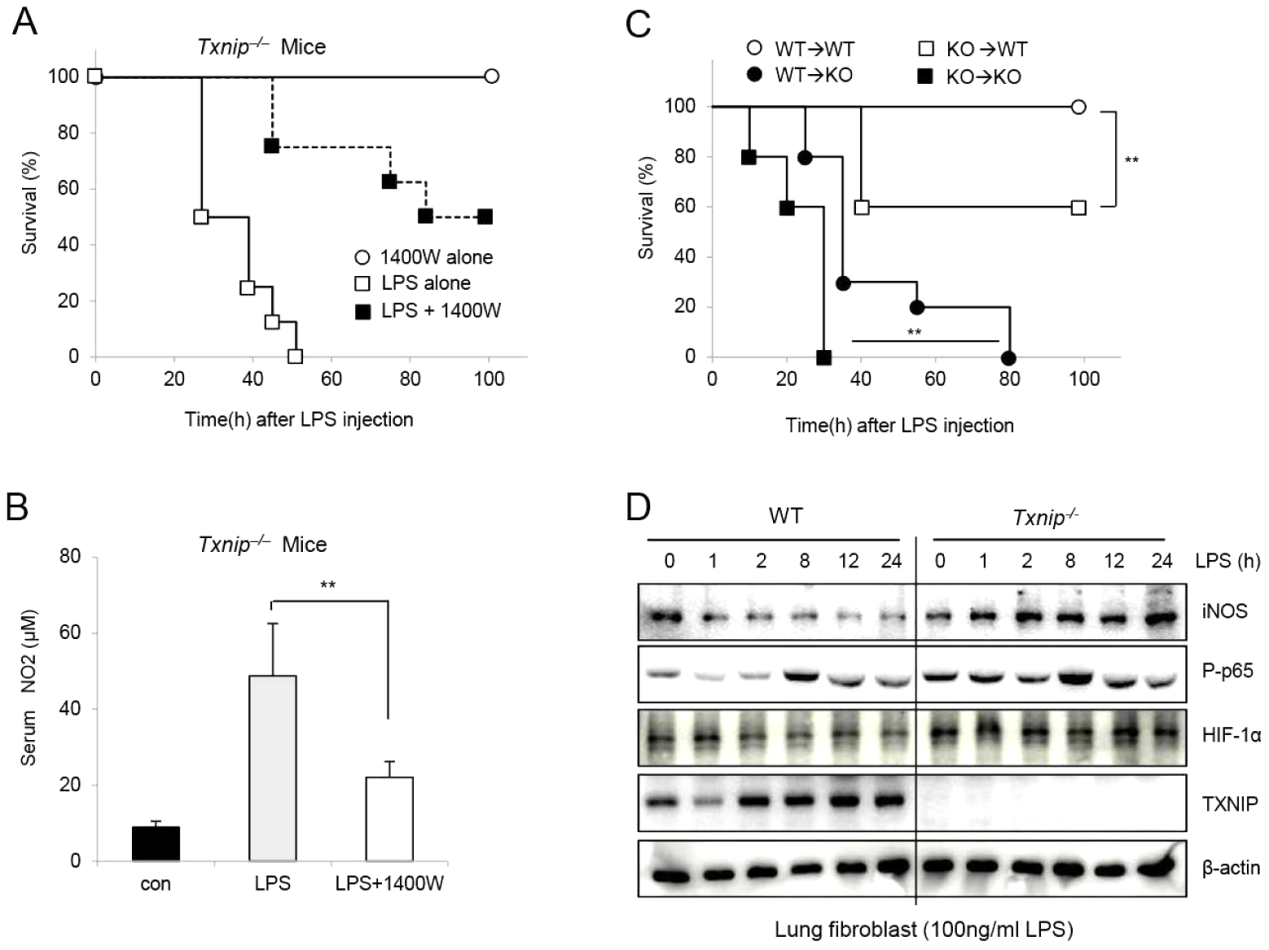
An iNOS inhibitor (1400W) rescues *Txnip^−/−^* mice injected with LPS *in vivo*. (A) 1400W (10 mg/kg body weight) was used to treat mice at 1 h prior to LPS (10 mg/kg body weight; i.p.) injection. Mice were divided into a 1400W group (n = 7), an LPS-alone group, and an LPS+1400W group (n = 8). Viability was assessed every 5 h. (B) The concentrations of serum NO in *Txnip^−/−^* mice (n = 5 per group) were determined. After LPS injection, the serum concentrations of NO were significantly affected by 1400W treatment. These data represent repeated results from at least 3 independent experiments (**P<0.01). (C) WT or *Txnip^−/−^* mice were BM-transplanted as indicated, and survival rates were determined after LPS (10 mg/kg; i.p.) treatment (**P<0.01). (D) Lung fibroblasts from WT or *Txnip^−/−^* mice were treated with 100 ng/ml LPS, and the cell lysates were harvested at the indicated times. Western blot analysis was performed using iNOS, phospho-p65, HIF-1α, and TXNIP antibodies. The detection of β-actin in each sample served as a loading control. The data are representative of at least 3 repeated experiments.

To confirm that the increase in NO release after LPS injection was contributing to the death of the *Txnip^−/−^* mice, we determined whether the inhibition of NO production by an iNOS inhibitor (1400W) would protect *Txnip^−/−^* mice from endotoxic shock-induced death. Indeed, 1400W treatment rescued approximately 50% of the *Txnip^−/−^* mice that were injected with LPS (Fig. 4 A), and similar results were observed using L-NAME, another inhibitor of iNOS (Fig. S2 A–C). These findings demonstrate that the inhibition of NO release by iNOS inhibitors reduced the increased sensitivity of *Txnip^−/−^* mice to LPS. To further investigate this finding, we examined whether 1400W could prevent NO production in the serum of *Txnip^−/−^* mice after LPS injection. Eighteen hours post-LPS treatment, the concentration of NO in the serum was significantly decreased in mice treated with 1400W (Fig. 4 B).

We also examined whether the death of *Txnip^−/−^* mice observed after LPS injection was a direct result of the absence of TXNIP in immune cells by generating BM chimeras. When the WT recipient mice were transplanted with *Txnip^−/−^* BM, the survival rate after LPS injection was lower than the survival rate of mice transplanted with WT BM. In contrast, the survival rate of *Txnip^−/−^* mice transplanted with WT BM was greater than that of *Txnip^−/−^* mice transplanted with *Txnip^−/−^* BM (Fig. 4 C). However, the transplantation of WT BM into *Txnip^−/−^* mice did not reduce the mortality to the rate observed when WT BM was transplanted into WT mice, suggesting that the mortality in response to LPS injection was dependent on donor BM cells and the host microenvironment. To confirm the regulation of iNOS, NF-κB and HIF-1α expression by TXNIP in nonimmune cells, we assessed the expression of these factors in lung fibroblasts from WT and *Txnip^−/−^* mice in response to LPS treatment (Fig. 4 D). We observed results in lung fibroblasts similar to those in immune cells (macrophages and neutrophils), and these results confirmed that TXNIP regulates the iNOS pathway similarly in immune and nonimmune cells. Overall, these results suggest that TXNIP plays a critical role as a negative modulator of NO and iNOS expression during LPS-induced endotoxic shock.

### TXNIP negatively regulates the production of NO and iNOS expression in response to LPS

**Figure 5:**
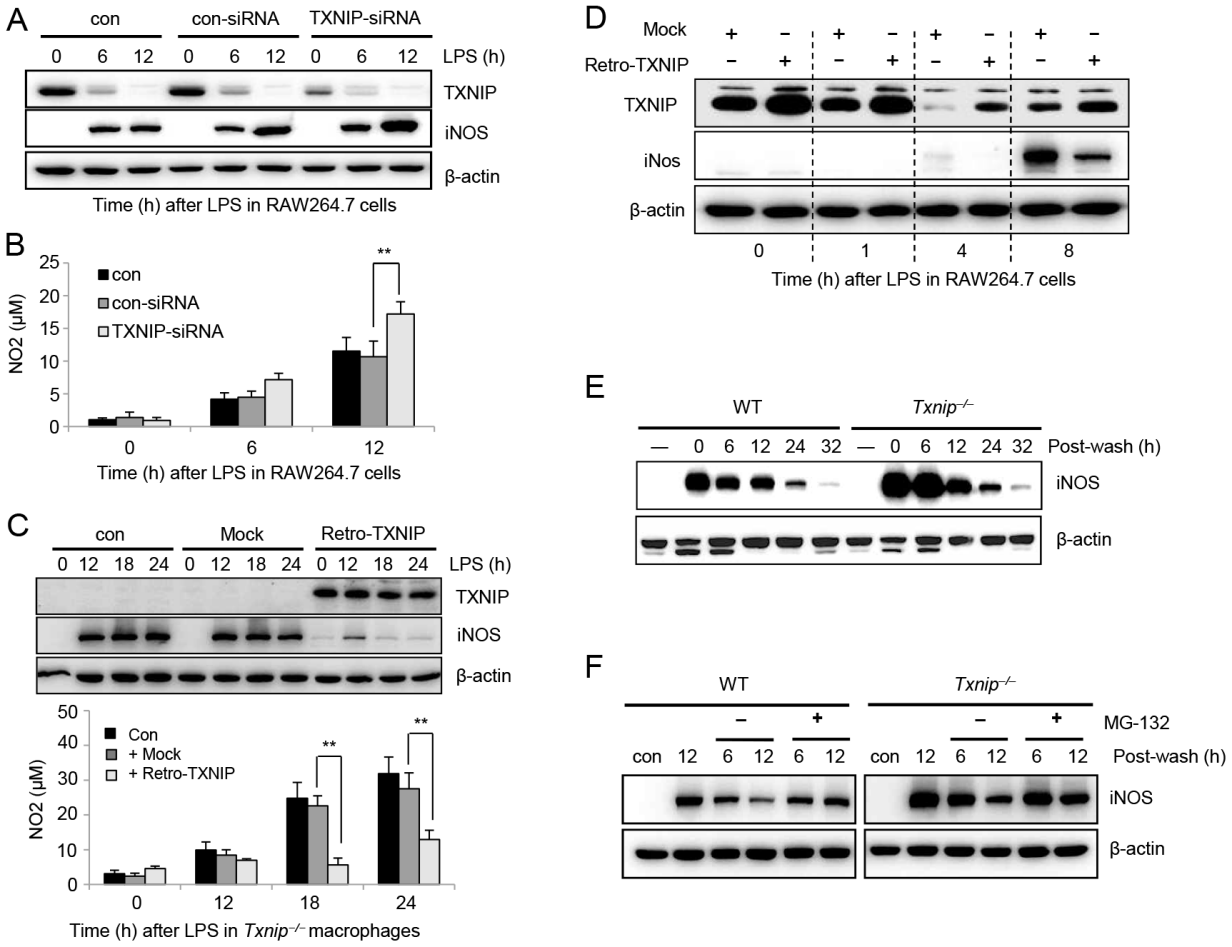
TXNIP negatively regulates the production of NO and iNOS expression in response to LPS. (A) RAW264.7 cells were transfected with control siRNA and TXNIP siRNA. At 48 h post-transfection, cells were treated with 100 ng/ml LPS for 6 or 12 h and then harvested. The expression of iNOS and TXNIP in RAW264.7 cells was measured by western blot. (B) NO production in response to LPS was determined by measuring the amount of nitrite in the culture media using Griess reagents. Data are presented as the mean ± SD of 3 independent experiments (**P<0.01). (C) *Txnip^−/−^* macrophages were infected with TXNIP retrovirus or control retrovirus at 200 plaque-forming units (PFU) per cell, and the cells were incubated in the presence or absence of 100 ng/ml LPS at the indicated times. The expression of TXNIP and iNOS was measured by western blot (top). NO production from the macrophages was determined by measuring the amount of nitrite in the culture media using Griess reagents (bottom). Data are presented as the mean ± SD of 3 independent experiments (**P<0.01). (D) For the overexpression of TXNIP, RAW264.7 cells were infected with TXNIP retrovirus or control retrovirus at 200 PFU/cell, and the cells were incubated in the presence or absence of 100 ng/ml LPS at the indicated times. The expression of TXNIP and iNOS was measured by western blot. Data are representative of at least 3 repeated experiments. (E) Macrophages from WT or *Txnip^−/−^* mice were incubated with or without LPS overnight, washed, (F) replenished with fresh medium with (+) or without (−) 10 µM of the proteasomal inhibitor MG-132, and then lysed at the indicated time points after washing. Western blot analysis was conducted using an anti-iNOS antibody, and the detection of actin in each sample serves as a loading control. Data are representative of at least 3 repeated experiments.

The above data demonstrate that both iNOS expression and NO production were dramatically increased in response to LPS stimulation in *Txnip^−/−^* mice. To understand molecular mechanisms of TXNIP-mediated NO regulation, we knocked down TXNIP expression in RAW264.7 cells using small interfering RNA (siRNA), and then we determined the change of iNOS expression and NO production after LPS treatment. As shown in Figure 5A, TXNIP-siRNA, but not control siRNA, effectively reduced the levels of endogenous TXNIP protein. The silencing of TXNIP resulted in an increase in the expression of iNOS, and accordingly, NO production was increased to a certain degree in TXNIP siRNA-transfected RAW264.7 cells after LPS stimulation compared to that observed in control-siRNA-transfected cells (Fig. 5 A and B).

Furthermore, to investigate whether the regulation of iNOS expression by TXNIP was directly related to the increased production of NO in *Txnip^−/−^* cells in response to LPS stimulation, TXNIP expression was restored in *Txnip^−/−^* macrophages by transducing a TXNIP-expressing retrovirus. When TXNIP expression was restored in *Txnip^−/−^* macrophages, the expression of iNOS and the production of NO was decreased (Fig. 5 C), confirming that TXNIP has a direct role in the regulation of iNOS expression and the production of NO in response to LPS. Similarly, when TXNIP was overexpressed in RAW264.7 cells, the levels of iNOS were lower than those of the control transfectants in response to LPS treatment, further confirming the direct regulation of iNOS expression by TXNIP (Fig. 5 D). To determine whether NO contributes to the expression or degradation of TXNIP, we examined the stability of TXNIP in the presence of a NO donor, DETA-NONOate. However, DETA-NONOate had no effect on the expression or degradation of TXNIP (Fig. S3).

iNOS has been shown to be ubiquitinated by the E3 ubiquitin ligase complex and degraded in various cell lines [21], [22]. To determine whether the expression or stability of iNOS was altered by TXNIP, macrophages from WT and *Txnip^−/−^* mice were stimulated with LPS for 12 h and washed to remove the LPS. Then, iNOS expression was monitored at various times (Fig. 5 E). The initial expression of LPS-induced iNOS was significantly elevated in *Txnip^−/−^* macrophages, although the kinetics of degradation were similar in both types of cells. In addition, to determine whether TXNIP regulated the degradation of iNOS via the proteasomal pathway, WT and *Txnip^−/−^* macrophages were treated with 100 ng/ml of LPS plus MG-132, a proteasome inhibitor (Fig. 5 F). As the MG-132-mediated inhibition of iNOS degradation was similar in both WT and *Txnip^−/−^* macrophages, the degradation of iNOS by the proteosome is likely not dependent on TXNIP. Combined, these results suggest that TXNIP has a role in the induction of iNOS expression but not in the degradation of iNOS.

### TXNIP negatively modulates NF-κB activation in response to LPS

**Figure 6:**
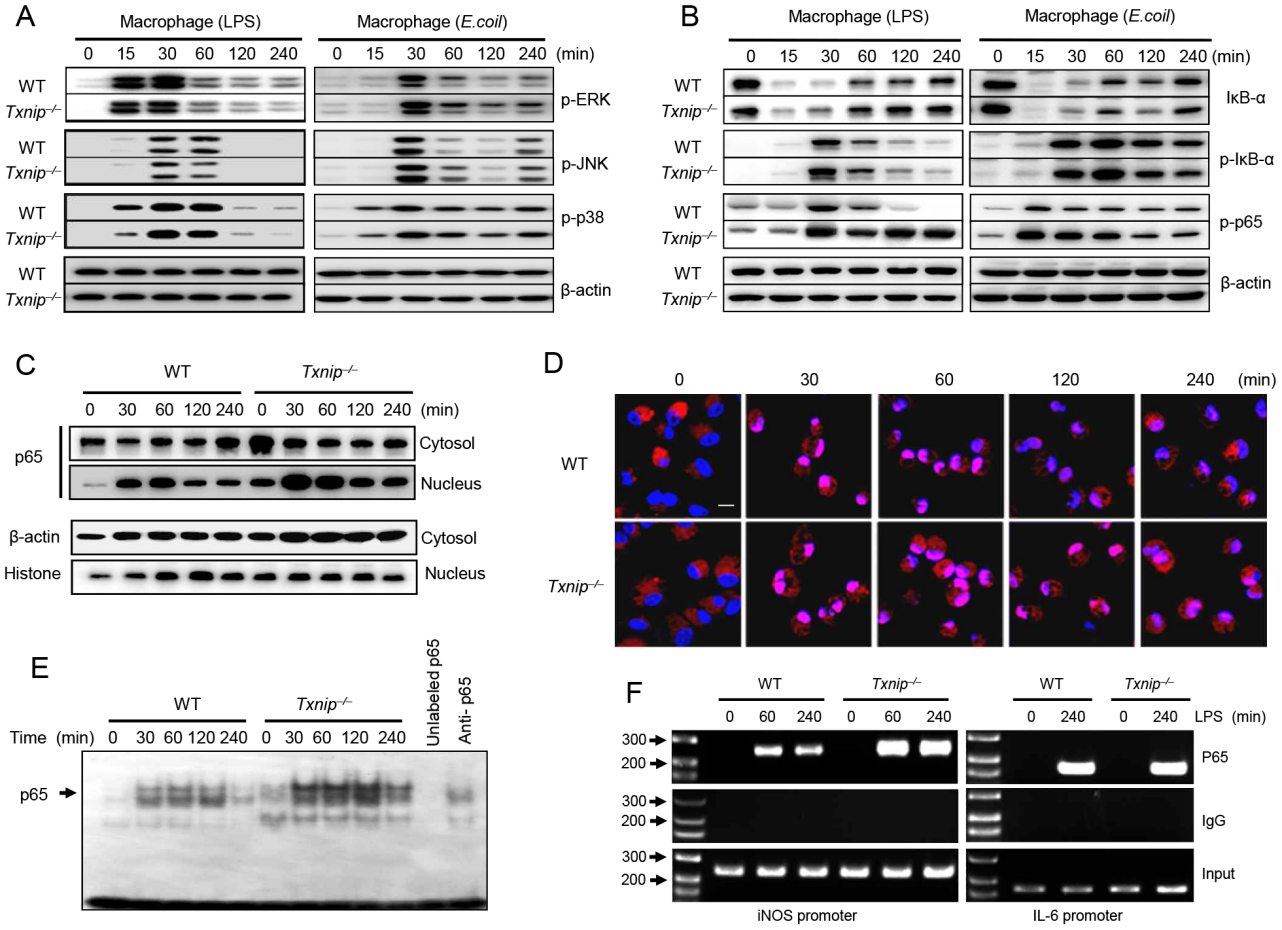
TXNIP deficiency promotes NF-kB activation mediated by LPS or *E. coli* stimulation. Peritoneal macrophages from WT or *Txnip^−/−^* mice were treated with 100 ng/ml LPS or *E. coli* at a MOI of 10, and cell lysates were harvested at the indicated times. (A) The levels of phosphorylated ERK, JNK, and p38 were determined by western blotting. The same blots were stripped and reprobed with an anti–β-actin antibody. (B) The experimental conditions followed the pattern outlined in (A). Western blot analysis was performed using anti-IκBα, anti–phospho-IκBα, and anti–phospho-p65 antibodies. The detection of β-actin in each sample serves as a loading control. The data are representative of at least 3 repeated experiments. (C) Cytosolic fractions and nuclear extracts were prepared from macrophages treated with 100 ng/ml LPS at the time points indicated. The NF-κB p65 levels were determined by western blot analysis. The detection of β-actin in cytosolic fractions and histone H1 in nuclear extracts served as a loading control. The data are representative of at least 3 repeated experiments. (D) The nuclear translocation of NF-κB was evaluated by immunohistochemistry using an antibody for the NF-κB p65 subunit (stained with DAPI (blue) and conjugated to the red fluorescent dye Alexa Fluor 546 (Alexa546)). LPS-treated cells were fixed, permeabilized, and analyzed by confocal microscopy (Carl Zeiss, LSM510). Scale bar, 20 µm. (E) Nuclear extracts (10 µg) of isolated macrophages from WT and *Txnip^−/−^* mice were analyzed by EMSA. The specificity of NF-κB binding was assessed by incubating the nuclear extracts with a 100-fold excess of unlabeled specific probe. (F) Peritoneal macrophages from WT or *Txnip^−/−^* mice were stimulated with LPS, and the cells were processed for ChIP assays at the indicated time points. The antibodies used for the ChIP assays are shown on the right. The input indicates the control.

It has been reported that treatment with LPS can induce the rapid phosphorylation of MAPKs (e.g., JNK, ERK1/2, and p38) in macrophages and neutrophils [23]. Therefore, we examined whether TXNIP affects MAPK signaling after LPS or *E. coli* treatment. There were no significant differences in MAPK phosphorylation between WT and *Txnip^−/−^* macrophages or neutrophils (Fig. 6 A, left and Fig. S4 A), indicating that TXNIP is not involved in the activation of MAPKs in response to LPS treatment.

After LPS administration, NF-κB translocates into the nucleus, where it binds to the promoters of target genes to activate transcription, and we previously detailed the role of TXNIP in the regulation of NF-κB activity. It has been demonstrated that TXNIP downregulates TNF-α-induced NF-κB activity by interacting with HDAC1 and HDAC3 [24]. Therefore, we examined the role of TXNIP in the regulation of LPS or *E. coli*-induced NF-κB activation in peritoneal macrophages from WT and *Txnip^−/−^* mice. The phosphorylation of p65 was dramatically increased in *Txnip^−/−^* macrophages after treatment with LPS for 30 min, and this increase was maintained in *Txnip^−/−^* macrophages until 240 min post-treatment. Interestingly, IκB-α degradation and IκB-α phosphorylation were similar in WT and *Txnip^−/−^* macrophages after LPS stimulation (Fig. 6 B). To determine if these results were a cell type-specific effect, BM neutrophils isolated from WT or *Txnip^−/−^* mice were treated with 100 ng/ml LPS. Similar results were observed when neutrophils were treated with LPS, demonstrating that this effect was not cell type-specific (Fig. S4 B).

Next, we examined the nuclear translocation of NF-κB. As shown by western blot analysis, the increased nuclear p65 levels and decreased cytosolic p65 levels in *Txnip^−/−^* macrophages were maintained up to 240 min after LPS treatment. In contrast, p65 was mostly present in the cytosol of WT macrophages up to 120 min after LPS treatment (Fig. 6 C). In support of this expression pattern, using immunocytochemical analysis, we identified a significant increase in the nuclear translocation of NF-κB p65 in LPS-treated *Txnip^−/−^* macrophages compared to WT macrophages, and this translocation was maintained until 240 min after treatment in *Txnip^−/−^* macrophages (Fig. 6 D). Furthermore, we investigated the DNA-binding activity of NF-κB in macrophages from WT and *Txnip^−/−^* mice following LPS stimulation using electrophoretic mobility shift assays (EMSAs). The NF-κB DNA-binding activity in nuclear extracts from LPS-stimulated *Txnip^−/−^* macrophages was increased compared to that observed in WT macrophages (Fig. 6 E).

To evaluate whether altered NF-κB activity in *Txnip^−/−^* macrophages could alter endogenous NF-κB promoter responses, the direct complex formation between NF-κB and iNOS chromatin was examined by chromatin immunoprecipitation (ChIP) assay using antibodies to the NF-κB p65 subunit or IgG. NF-κB p65 binding to the iNOS promoter was increased in *Txnip^−/−^* macrophages compared to WT macrophages in response to LPS stimulation for 1 h (Fig. 6 F). These data are in agreement with our real-time PCR results indicating the upregulation of iNOS expression in *Txnip^−/−^* cells (Fig. 3 B). Because we had observed that TNF-α and IL-6 were increased to similar levels in both groups of mice (Fig. 2), ChIP assays were performed on the IL-6 promoter for the NF-κB p65 subunit, and we found that NF-κB p65 binding to the IL-6 promoter was similar in *Txnip^−/−^* macrophages and WT macrophages in response to LPS stimulation for 1 h (Fig. 6 F). Overall, these data indicate that LPS-induced iNOS activation appears to be mediated predominantly by the NF-κB pathway in the absence of TXNIP.

### Excessive NO produced by *Txnip^−/−^* cells acts as a negative regulator of the activation of the NLRP3 inflammasome

**Figure 7:**
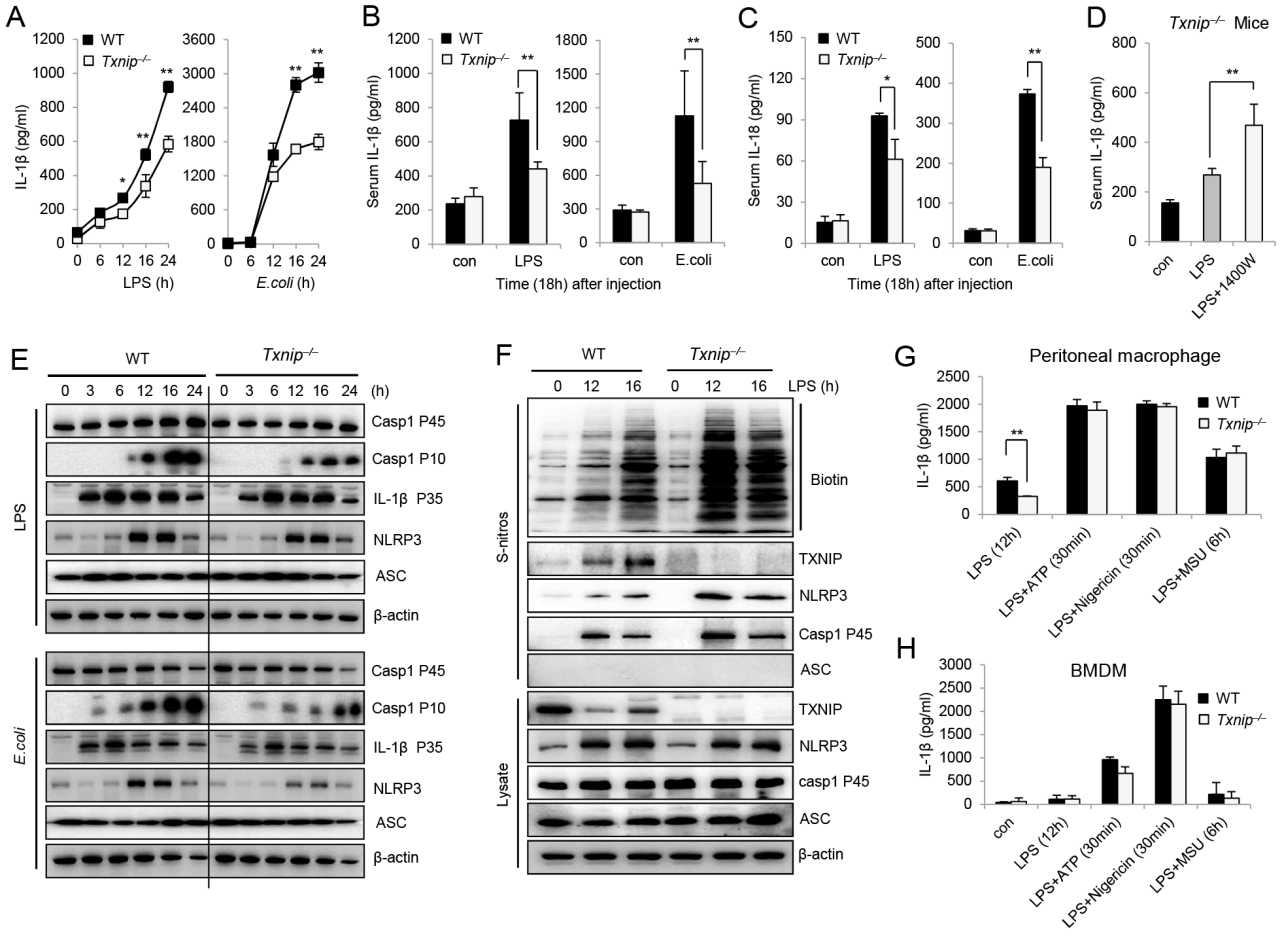
TXNIP deficiency-induced NO inhibits activation of the NLRP3 inflammasome via S-nitrosylation. (A) Peritoneal macrophages from WT or *Txnip^−/−^* mice were treated with 100 ng/ml LPS and *E. coli* at a MOI of 10. The supernatants were harvested at the indicated times. The levels of IL-1β in the culture supernatants were measured by ELISA. (B and C) After LPS or *E. coli* injection in WT (n = 5 per) and *Txnip^−/−^* mice (n = 5 per), the levels of serum IL-1β (B) and IL-18 (C) were measured by ELISA. Bacterial peritonitis was induced by the i.p. injection of 10^8^ CFU of live *E. coli* (DH5α). (D) Concentrations of serum IL-1β in *Txnip^−/−^* mice (n = 5 per group) were determined. After LPS injection, the serum concentrations of NO were significantly altered by 1400W treatment. These data represent at least 3 independent experiments (*P<0.05, **P<0.01). (E) Immunoblot analysis of inflammasome components, including caspase-1, NLRP3, and ASC, in WT and *Txnip^−/−^* macrophages at the indicated time points. (F) The total level of S-nitrosylation of inflammasome components was determined by immunoblot analysis. LPS was used to treat macrophages from WT and *Txnip^−/−^* mice at the indicated time points. Below (lysate), immunoblot analysis of total lysate fractions. Data are representative of at least 3 repeated experiments. Peritoneal macrophages (G) and BMDMs (H) from WT and *Txnip^−/−^* mice were primed with LPS for 12 h and then stimulated with inflammasome activators such as ATP (5 mM) for 30 min, nigericin (10 µM) for 30 min, or MSU (200 µg/ml) for 6 h. IL-1β secretion was measured in the culture supernatants by ELISA. Data are presented as the mean ± SD of 3 independent experiments (**P<0.01).

The dramatic enhancement of both NO and the expression of iNOS in *Txnip^−/−^* macrophages suggested that TXNIP may be involved in NO-mediated NLRP3 inhibition. To test this hypothesis, we compared IL-1β production and S-nitrosylation of the NLRP3 inflammasome in peritoneal macrophages from WT and *Txnip^−/−^* mice. When cells were treated with LPS or *E. coli*, the secretion of mature IL-1β was decreased in *Txnip^−/−^* macrophages compared to WT macrophages (Fig. 7 A). We next analyzed the induction of pro-IL-1β mRNA levels after treatment with LPS in both WT and *Txnip^−/−^* macrophages (Fig. S5 A). The sera from *Txnip^−/−^* mice injected with LPS or *E. coli* displayed decreased levels of IL-1β as compared to the samples from WT mice (Fig. 7 B), and similar results were observed for the secretion of IL-18 (Fig. 7 C). Moreover, the serum concentration of IL-1β was increased in mice treated with 1400W (Fig. 7 D), suggesting that inflammasome-mediated IL-1β secretion is regulated by NO. Next, we assessed the levels of caspase-1 (P45 and P10), pro-IL-1β, NLRP3, and ASC in cell lysates after LPS or *E. coli* treatment. The active forms of caspase-1 were detected to a greater extent in WT cells, whereas the levels of pro-IL-1β, NLRP3, and ASC were not appreciably different in WT or *Txnip^−/−^* macrophages (Fig. 7 E).

After stimulating macrophages with LPS or *E. coli*, we analyzed the S-nitrosylation of NLRP3 inflammasome components including TXNIP, NLRP3, ASC, and caspase-1. The S-nitrosylation of NLRP3 and caspase-1 was significantly increased in *Txnip^−/−^* macrophages in response to LPS (Fig. 7 F) or *E. coli* treatment (Fig. S5 B). In addition, we analyzed NLRP3 nitrosylation after treatment with LPS in both WT and *Txnip^−/−^* BM-derived macrophages (BMDMs) and found that S-nitrosylation of NLRP3 and caspase-1 was increased to a greater extent in *Txnip^−/−^* BMDMs in response to LPS or *E. coli* (Fig. S5 C). Masters *et al.* tested NRLP3 inflammasome activation in BMDMs and found no difference in IL-1β secretion in response to IAPP or other inflammasome activators, such as MSU or ATP, in BMDMs [25]. Thus, we next examined NLRP3 activation by NLRP3 agonists such as ATP, Nigericin, and MSU in WT and *Txnip^−/−^* peritoneal macrophages or BMDMs and found that *Txnip^−/−^* macrophages displayed reduced IL-1β secretion with LPS stimulation alone. However, we observed no difference in IL-1β secretion in response to treatment with inflammasome activators in either peritoneal macrophages or BMDMs (Fig. 7 G and F).

Considering these results together, we hypothesized that *Txnip^−/−^* macrophages produced increased levels of NO following LPS treatment and that the NO induced by LPS priming may mediate the observed inhibitory effect. However, NLRP3 agonists induced higher levels of IL-1β secretion in both WT and *Txnip^−/−^* peritoneal macrophages and BMDMs. In addition, we evaluated the cytokine (Fig. S6 A) and protein (Fig. S6 B) profiles of long-term LPS-stimulated macrophages, and based on these results, we propose that S-nitrosylation of the NLRP3 inflammasome, caused by excessive NO resulting from the absence of TXNIP, may be responsible for the inhibition of IL-1β processing. Overall, these results suggest that excessive NO produced by *Txnip^−/−^* cells could inhibit NLRP3 inflammasome activation. Furthermore, our *in vivo* results support the hypothesis that excessive NO induced by LPS treatment in *Txnip^−/−^* mice inhibited NLRP3 inflammasome activation and then prevented the secretion of IL-1β.

**Figure 8:**
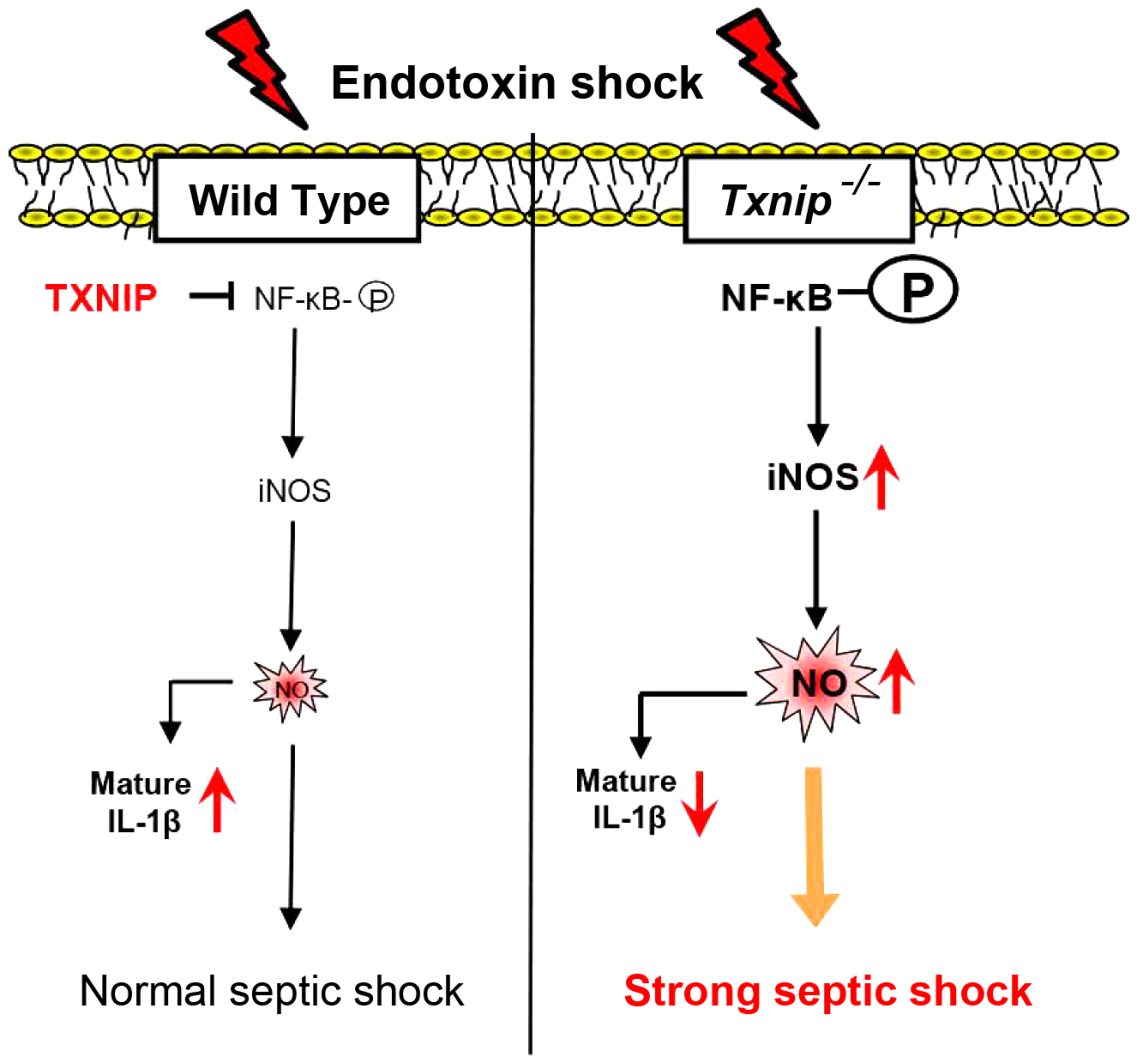
A schematic model showing TXNIP-mediated regulatory pathways for LPS-mediated inflammatory signaling. TXNIP-iNOS-NO is a main pathway involved in the generation of septic shock. The excessive NO production in *Txnip^−/−^* mice reduced the production of IL-1β by inhibiting the NLRP3 inflammasome.

Based on these observations, a schematic model was proposed to delineate the roles of TXNIP in LPS-mediated inflammatory signaling (Fig. 8). In this model, the absence of TXNIP induces excessive NO production, which is the main driving force in endotoxic shock. During this process, TXNIP regulates the NF-κB, which affects the iNOS pathway in response to LPS treatment. In addition, excessive NO inhibits NLRP3-dependent IL-1 secretion via direct S-nitrosylation of the NLRP3 inflammasome.

## Discussion

TXNIP has many biological functions, including the inhibition of tumor growth, suppression of hepatocarcinogenesis, and regulation of glucose metabolism and ROS generation in different cell types [26]–[30]. However, to the best of our knowledge, the function of TXNIP in endotoxic shock has not been previously examined. Here, we demonstrate a novel role for TXNIP in the modulation of the inflammatory response after LPS treatment. The following observations support this role: (i) the extreme sensitivity of *Txnip^−/−^* mice to LPS-induced mortality, with reduced blood glucose levels and a reduced body temperature; (ii) the increased NO production and iNOS expression in *Txnip^−/−^* mice; (iii) the increased phosphorylation and nuclear translocation of NF-κB p65 in *Txnip^−/−^* macrophages; and (iv) the increased S-nitrosylation of NLRP3 inflammasome components and decreased IL-1β maturation in *Txnip^−/−^* macrophages.

Initially, we hypothesized that TXNIP may be involved in the inflammatory response via alterations in proinflammatory mediators such as cytokines, MAPKs, ROS, or NF-κB. Unexpectedly, there were no significant differences in the production of inflammatory cytokines or MAPK phosphorylation in WT and *Txnip^−/−^* macrophages after LPS treatment.

Previous studies have demonstrated that TXNIP interacts with the antioxidant thioredoxin to inhibit its reducing activity [2], [4]. However, our data indicate that the overall levels of H_2_O_2_ and superoxide during LPS-induced inflammation were elevated in *Txnip^−/−^* macrophages compared to WT cells. In addition, another group failed to observe significant changes in the amount of available thioredoxin in the absence of TXNIP [31]. These results suggest that when the physiological level of TXNIP is not high enough to bind thioredoxin, oxidized TXNIP is easily released from thioredoxin, and TXNIP-thioredoxin binding may be restricted to the nucleus or mitochondria after LPS stimulation [32].

One recent study showed that TXNIP expression driven by MondoA:Mlx was rapidly suppressed by various inflammatory stimuli such as LPS [33]. Our data also demonstrated that TXNIP expression was dramatically decreased after LPS treatment, although this expression was gradually restored after 16 h. In addition, iNOS expression began to increase at 12 h post-LPS treatment, suggesting the possibility that the downregulation of TXNIP may be a prerequisite for full induction of iNOS by LPS. At a minimum, the reduced expression of TXNIP likely provides favorable conditions for iNOS induction by LPS. The NF-κB protein complex controls the transcription of specific genes and is involved in cellular responses to various stimuli, including stress, cytokines, free radicals, and bacterial or viral antigens.

Histone deacetylase 1 (HDAC1) interacts with NF-κB p65 and can suppress NF-κB activity [34]. We previously reported that TXNIP may serve as a component of the complex that forms between HDAC1 and NF-κB p65, as both TXNIP and HDAC1 could suppress NF-κB activity [24]. In this previous study, we observed that TXNIP inhibited CBP (CREB-binding protein)-induced NF-κB activation and p65 acetylation. Moreover, TXNIP could bind to HDAC1 and HDAC3 and co-localize with HDAC1 and HDAC3, and NF-κB activation by CBP was synergistically decreased by HDAC1, HDAC3, and TXNIP.

Based on these observations, we hypothesized that TXNIP interacts with HDACs and that residue 247 of TXNIP is crucial for the HDAC interaction to suppress NF-κB activity. Furthermore, we demonstrated that TXNIP represses the phosphorylation and nuclear translocation of NF-κB p65, as the phosphorylation and nuclear translocation of p65 was substantially upregulated in *Txnip^−/−^* macrophages. Because significant changes were not observed in either MAPK components or IκB-α after LPS treatment, these data suggest that TXNIP is specifically involved in the regulation of NF-κB phosphorylation and translocation, independent of the activation of inflammatory cytokines. This phenomenon constitutes a novel immunoregulatory mechanism that could potentially be targeted in the development of new therapeutic approaches to treat inflammatory diseases.

We previously reported that TXNIP is part of the pVHL/HIF-1α complex, which negatively regulates HIF1α, and that TXNIP plays a role in the CRM1-dependent nuclear export of this complex. The induction of the nuclear export regulatory function of TXNIP is one of key mechanisms responsible for the degradation of HIF-1α during cell stress conditions [35]. As demonstrated previously, NF-κB is a critical transcriptional activator of HIF-1α, and basal NF-κB activity is required for the accumulation of the HIF-1α protein [36]–[38], and one recent study reported that HIF-1α is critical for the LPS driven transcription of IL-1β [39]. Our data demonstrate that both the phosphorylation and translocation of p65 and the accumulation of HIF-1α in *Txnip^−/−^* macrophages were dramatically increased after LPS stimulation in comparison to WT macrophages (unpublished data).

Recently, it has been demonstrated that both NF-κB and HIF-1α enhance iNOS expression in a variety of cell types [40]–[42], and it was reported that exogenous and endogenous NO can suppress TXNIP expression and that TXNIP facilitates nitrosative stress [20]. However, the direct effects of TXNIP on NO regulation in LPS-induced endotoxic shock were not investigated. In the current study, the importance of the overproduction of NO for the observed sensitivity to endotoxic shock was demonstrated via the use of NO synthase inhibitors. 1400W treatment of *Txnip^−/−^* mice with septic shock reduced the blood NO levels and inhibited endotoxin-induced death. Thus, we believe there is cross-talk between the TXNIP and the NF-κB/iNOS pathways and that this interaction may regulate disease states associated with sepsis.

In addition, we observed that excessive NO due to the absence of TXNIP regulated the activation of the NLRP3 inflammasome. Thus, we hypothesized that S-nitrosylation of NLRP3 by NO would not only affect the reduction of IL-1β but also the sensitivity to lethal endotoxin-induced shock. According to our data (shown in Fig. 1, B and C), the blood glucose level and body temperature of *Txnip^−/−^* mice were significantly lower than those of WT mice after LPS injection. NO is known as a pivotal mediator in the development of hypoxia-induced hypothermia [43]. Furthermore, in patients with sepsis, caspase-1 levels are significantly decreased, and the inhibition of caspase-1 and defective IL-1β production constitute an important immunological feature of sepsis [44]. Since increased IL-1β production makes the mice more susceptible to LPS injection, it seems that decreased IL-1β production is not involved in the increased susceptibility of *Txnip^−/−^* mice.

In summary, our results demonstrate that TXNIP plays a critical role in the control of lethal endotoxin-induced shock by controlling NO production in innate immune cells via the regulation of iNOS expression. This regulation is mediated through changes in the activation and translocation of NF-κB that affect the NF-κB/iNOS pathway. In addition, excess levels of NO inhibit IL-1β production via S-nitrosylation of the NLRP3 inflammasome. Subsequently, the survival of *Txnip^−/−^* mice was significantly decreased due to hypothermia and hypoglycemia. Overall, the elucidation of this pathway identifies a novel therapeutic target for the treatment of inflammatory diseases.

## Materials and Methods

### Mice and reagents

All animal-related procedures were reviewed and approved by the Institutional Animal Care and Use Committee of the Korea Research Institute of Bioscience and Biotechnology (KRIBB-IACUC, approval number: KRIBB-AEC-11044), and all procedures were performed in accordance with institutional (National Institutes of Health, USA) guidelines for animal care. WT C57BL/6 mice were obtained from Koatech, and mice with a targeted deletion of TXNIP (homozygous mice and their homozygous littermates) were generated as previously described [45]. All mice were housed in a pathogen-free animal facility under a standard light-dark cycle with standard rodent chow and water provided *ad libitum*. The experimental groups were age- and sex-matched. Mice were injected intraperitoneally (i.p.) with 10 mg/kg of LPS (Sigma-Aldrich) at 10–12 weeks of age. For sepsis models with a single bacterial species, bacterial peritonitis was induced by i.p. injection of 10^8^ colony-forming units (CFU) of live *E. coli* (DH5α). 1400W and L-NAME were purchased from Sigma-Aldrich. RAW264.7 cells were purchased from the American Type Culture Collection and grown in RPMI media supplemented with 10% FBS. Transient transfections were performed using Lipofectamine 2000 (Invitrogen), as recommended by the manufacturer. The siRNA specific for TXNIP (1299003) was purchased from Invitrogen. Cells were treated as indicated in the figures and processed for analysis by western blot and immunoprecipitation, as described previously (Yang *et al.*, 2007). For western blot analysis, antibodies specific for p-JNK (4668), JNK (9258), pNF-κB p65 (3033), NF-κB p65 (4764), p-Erk1/2 (4370), Erk1/2 (9102), p-p38 (9215), p38 (9212), p-IκB(2859), IκB (4812) and IL-1β (8689) were purchased from Cell Signaling Technology. Antibodies for iNOS (SC-650), ASC1 (sc-22514-R), Caspase-1 (sc-514), and β-actin (sc-47778) were purchased from Santa Cruz Biotechnology. NLRP3 (AG-20B-0014) antibody was purchased from Adipogen. TXNIP antibody was purchased from MBL International.

### Preparation of peritoneal macrophages, BMDMs and BM neutrophils

Peritoneal macrophages were harvested 4 days after the i.p. injection of 3% thioglycollate (Sigma). Macrophages were washed and plated in 24-well plates at 0.5×10^6^ cells per well. After incubation for 2 h at 37°C, the wells were washed 3 times to remove all nonadherent cells. Finally, the culture medium was replaced with RPMI supplemented with 10% fetal bovine serum, sodium pyruvate, nonessential amino acids, penicillin G (100 IU/ml), and streptomycin (100 µg/ml). BM from WT and *Txnip^−/−^* mice was isolated and cultured, as described previously [25]. For the preparation of BMDMs, BM was differentiated in 20% (vol/vol) L929 cell-conditioned medium for 7 days. The L929 cell-culture Dulbecco's modified Eagle's medium (DMEM) was supplemented with 10% fetal bovine serum. The mouse neutrophils were isolated from the BM using MACS separation CS columns (130-041-305, Miltenyi Biotec). BM cells were incubated with primary antibodies for the cell surface markers CD45R/B220 (553086 BD Biosciences), TER119(116204, Bio Legend), F4/80(122604, Bio Legend), CD4(100404, Bio Legend), and CD5(100604, Bio Legend) for 20 min at 4°C. After incubation, anti-biotin Microbeads (130-090-450, Miltenyi Biotec) were added. Finally, the cell suspension was loaded onto the MACS column. T cells, B cells, RBC, monocytes, and macrophages were captured in the column, allowing the neutrophils to pass through by negative selection. The neutrophils were then cultured in 24-well plates at 1×10^6^ cells per well.

### Real-time PCR and ELISA

Total RNA was extracted from macrophages using TRIzol reagent (Invitrogen), as recommended by the manufacturer. Total RNA (1 µg) was reverse-transcribed and analyzed using real-time PCR with a Dice TP 800 Thermal Cycler and SYBR Premix Ex Taq (Takara Bio). The mRNA expression level was calculated using HPRT as the control. The primer sequences were as follows: mouse iNOS, forward 5′-ACATCGACCCGTCCACAGTAT-3′ and reverse 5′-CAGAGGGGTAGGCTTGTCTC-3′; mouse TXNIP, forward 5′-TGTGAAGTTACCCGAGTCAAAGC-3′ and reverse 5′-AGCGCAAGTAGTCCAAAGTCT-3′; mouse IL-1β, forward 5′- TTCAAATCTCGCAGCAGCAC-3′ and reverse 5′- AGCTTCTCCACAGCCACAAT-3′; and mouse HPRT, forward 5′-GCCTAAGATGAGCGCAAGTTG-3′ and reverse 5′-TACTAGGCAGATGGCCACAGG-3′. For ELISA, serum, tissue, and culture media were analyzed for cytokine content using DuoSet antibody pairs (R&D Systems) for the detection of IL-1β, IL-18, TNF-α, and IL-6.

### Serum, tissue, and culture media nitrite determination

The concentration of nitrite in the serum, liver, lung, and spleen samples was determined using a NO (total) detection kit (ADI-917-020, Enzo Life Sciences). Liver, lung, and spleen tissue was harvested, immediately flash frozen, and homogenized in a buffer containing 1 mM protease inhibitor cocktail. NO detection in culture media was performed using Griess reagent (G4410, Sigma) at 540 nm.

### Detection of S-nitrosylated proteins

The detection of S-nitrosylated proteins was performed using an S-nitrosylated protein detection assay kit (10006518, Cayman), as recommended by the manufacturer. For immunoprecipitation, S-nitrosylated proteins were incubated with NeutrAvidin-agarose resin (29200, Thermo Scientific) for 30 min at room temperature. After 5 washes with washing buffer from the assay kit, the samples were centrifuged at 200× *g* for 1 min at room temperature. Finally, the detection of S-nitrosylated proteins was performed using western blot. Biotin was detected using the S-nitrosylation detection reagent 1-tagged HRP provided with the assay kit.

### Immunostaining

Cells were immunostained as previously described [46]. Peritoneal macrophages were plated on round glass cover slips in 24-well plates 24 h prior to treatment with 100 ng/ml LPS. After LPS treatment, the samples were washed with cold PBS and fixed in 4% paraformaldehyde in PBS for 20 min at room temperature. The cells were then permeabilized with 0.2% Triton X-100 in PBS for 15 min at room temperature and incubated overnight at 4°C with either anti-p65 antibody. After 3 washes with PBS, the cells were incubated with Alexa Fluor 546–conjugated goat-anti mouse IgG (Invitrogen) for 2 h at room temperature and then washed again with PBS. The images were captured using a LSM510 confocal microscope (Carl Zeiss).

### ChIP assay

ChIP assays were performed according to the protocol included with the ChIP assay kit (17-295, Millipore). WT and *Txnip* ^−/−^ peritoneal macrophages were primed with LPS for 1 h and then fixed with formaldehyde for 10 min. After sonication, the cell lysate was confirmed to have 200- to 1,000-bp DNA fragments. For immunoprecipitation, an antibody for NF-κB p65 (Cell Signaling Technology 4764) was used at a dilution of 1∶100 and incubated with the sonicated cell lysate overnight. Finally, the sample was incubated with a salmon sperm DNA/Protein A agarose slurry for 1 h at 4°C. After 3 washes in the washing buffer provided with the assay kit, 5 M NaCl was added to the samples, and the samples were then incubated at 65°C for 4 h. The primers used for PCR (35 cycles) of the iNOS or IL-6 promoters were as follows: iNOS forward, 5′-GTCCCAGTTTTGAAGTGACTACG-3′; iNOS reverse, 5′-GTTGTGACCCTGGCAGCAG-3′; IL-6 forward, 5′-CGATGCTAAACGACGTCACATTGTGCA-3′; and IL-6 reverse, 5′-CTCCAGAGCAGAATGAGCTACAGACAT-3′. These primers were used as previously reported [47], [48].

### EMSA

Nuclear extracts were prepared from mouse macrophages stimulated with or without LPS (100 ng/ml) using a nuclear extract kit (100946, Active motif). An EMSA was performed using an NF-kB (5′-AGTTGAG GGGACTTTCCCAGGC-3′) consensus oligonucleotide that specifically binds to NF-kB. Double-stranded oligonucleotides were end-labeled with 2.5 mM γ-[32P] ATP using T4 kinase (Takara Shuzo, Otsu, Japan) and purified using PROBER columns (iNtRON Bio, Seongnam, Korea). The radioactive oligonucleotides were incubated with the nuclear extracts (10 µg) for 20 min in a binding buffer containing 10 mM Tris-HCl (pH 7.5), 50 mM NaCl, 1 mM DTT, 1 mM EDTA, 5% glycerol, 1 mg of poly (dI-dC), and 1 mg of BSA at room temperature. The samples were loaded onto a 5% polyacrylamide gel in TBE buffer (45 mM Tris base, 45 mM boric acid, 1.25 mM EDTA) and subjected to electrophoresis at 10 V/cm at 4°C. The gels were then vacuum-dried and examined using autoradiography.

### Retroviral infection of macrophages

Mouse TXNIP was cloned into the pMYs-IRES-GFP retroviral vector (Cell Biolabs). Retrovirus was generated with the Platinum-E retroviral packaging cell line (Cell Biolabs). Macrophages from *Txnip^−/−^* mice were infected with the retrovirus in RPMI 1640 medium containing 10% FBS and 8 µg/ml polybrene. Overexpression of TXNIP in macrophages was confirmed by quantitative real-time PCR and western blot.

### BM transplantation

TXNIP chimeras were generated as previously described [49]. Briefly, *Txnip^−/−^* recipients received WT bone BM, and WT recipients received *Txnip^−/−^* BM transplants. As a control for the nonspecific effects of radiation, WT recipients also received WT BM cell transplants, and *Txnip^−/−^* recipients received *Txnip^−/−^* BM cell transplants. Ten weeks after BM transplantation, the mice were i.p. injected with LPS and observed for survival for 100 h.

### Preparation of primary fibroblast cultures from mouse lungs

Primary fibroblast preparation was performed as described previously. Briefly, lung tissue was excised from either WT or *Txnip^−/−^* mice. The tissue was then minced into 1-mm pieces, which were plated onto p100 dishes (25 pieces per plate). Fetal bovine serum was added dropwise over each tissue piece, and then the plates were incubated for 4 h at 37°C. Then, 2 ml of DMEM containing 10% fetal bovine serum and antibiotics was added to each plate. The plates were monitored every day until the fibroblasts reached confluence.

### Statistical analysis

The results are expressed as the mean ± standard deviation and were compared using the 2-tailed Student's *t*-test for paired samples. For nonparametric data, the results are expressed as the median ± quartiles and were compared using the Wilcoxon signed rank test. An overall P value of <0.05 was considered statistically significant.

## Supporting Information

Figure S1 **The expression levels of TXNIP after LPS administration** ***in vivo*** **.** Liver, lung, and spleen samples were obtained from WT and *Txnip^−/−^* mice at 0, 2, 4, 8, and 16 h after LPS administration. Tissue lysates were prepared by homogenizing treatment and were then analyzed by immunoblotting with an anti-TXNIP antibody. These data are representative of at least 3 independent experiments.
(TIF)

Figure S2 **An iNOS inhibitor (L-NAME) rescues** ***Txnip^−/−^*** **mice injected with LPS** ***in vivo*** **.** (A) L-NAME (30 mg/kg body weight) treatment was performed 1 h prior to LPS (10 mg/kg body weight; i.p.) injection (L-NAME group, n = 7 and LPS-alone and LPS+L-NAME group, n = 10). Animal viability was assessed every 5 h. The concentrations of serum NO (B) and IL-1β (C) in *Txnip^−/−^* mice (n = 5 per group) were determined. After LPS injection, the serum concentrations of NO and IL-1β were significantly altered by L-NAME treatment. These data are representative of at least 3 independent experiments (**P<0.01).
(TIF)

Figure S3 **TXNIP does not affect the production of NO by the NO donor DETA-NONOate.** Macrophages from WT or *Txnip^−/−^* mice were incubated with the NO donor DETA-NONOate at the indicated concentrations. The expression of TXNIP was measured by western blot with an TXNIP antibody. These data are representative of at least 3 independent experiments.
(TIF)

Figure S4 **Signaling molecules activated in neutrophils after LPS treatment.** BM neutrophils from WT or *Txnip^−/−^* mice were treated with 100 ng/ml LPS, and cell lysates were harvested at the indicated time points. (A) The levels of phosphorylated ERK, JNK, and p38 were determined by western blotting. (B) Western blot analysis was performed using anti-p65 and anti-IκBα antibodies. The detection of β-actin in each sample served as a loading control. Data are representative of at least 3 independent experiments.
(TIF)

Figure S5 **S-nitrosylation of inflammasome components on NO.** (A) IL-1β mRNA levels of macrophages were determined by real-time PCR analysis. Peritoneal macrophages from WT or *Txnip^−/−^* mice were incubated with LPS (100 ng/ml) for the indicated time periods. (B) The total level of S-nitrosylation of inflammasome components was determined by immunoblot analysis. *E. coli* was used to treat peritoneal macrophages from WT and *Txnip^−/−^* mice at the indicated time points. Below (lysate), immunoblot analysis of total lysate fractions. Data are representative of at least 3 repeated experiments. (C) Total S-nitrosylation of inflammasome components, including NLRP3, caspase-1, and TXNIP was determined by immunoblot analysis. LPS or *E. coli* (MOI 10) was used to treat BMDMs from WT and *Txnip^−/−^* for the indicated time periods. Data are representative of at least 3 repeated experiments.
(TIF)

Figure S6 **TXNIP affects the production of IL-1β and iNOS following LPS treatment in macrophages.** Peritoneal macrophages from WT or *Txnip^−/−^* mice were treated with 100 ng/ml LPS, and the supernatants and cell lysates were harvested at the indicated time points (long-term). (A) The levels of IL-1β, TNF-α, and IL-6 in the culture supernatants were measured by ELISA. (B) Immunoblot analysis of iNOS, TXNIP, NLRP3, caspase-1, and ASC protein expression in macrophages. β-actin served as the loading control. Data are presented as the mean ± SD of 3 independent experiments (*P<0.05; **P<0.01)
(TIF)
