# Supplementary figures and images for: TXNIP Deficiency Exacerbates Endotoxic Shock via the Induction of Excessive Nitric Oxide Synthesis
Source: PLoS Pathog. 2013 Oct 3;9(10):e1003646. doi: 10.1371/journal.ppat.1003646 (PMC3789754; doi:10.1371/journal.ppat.1003646)

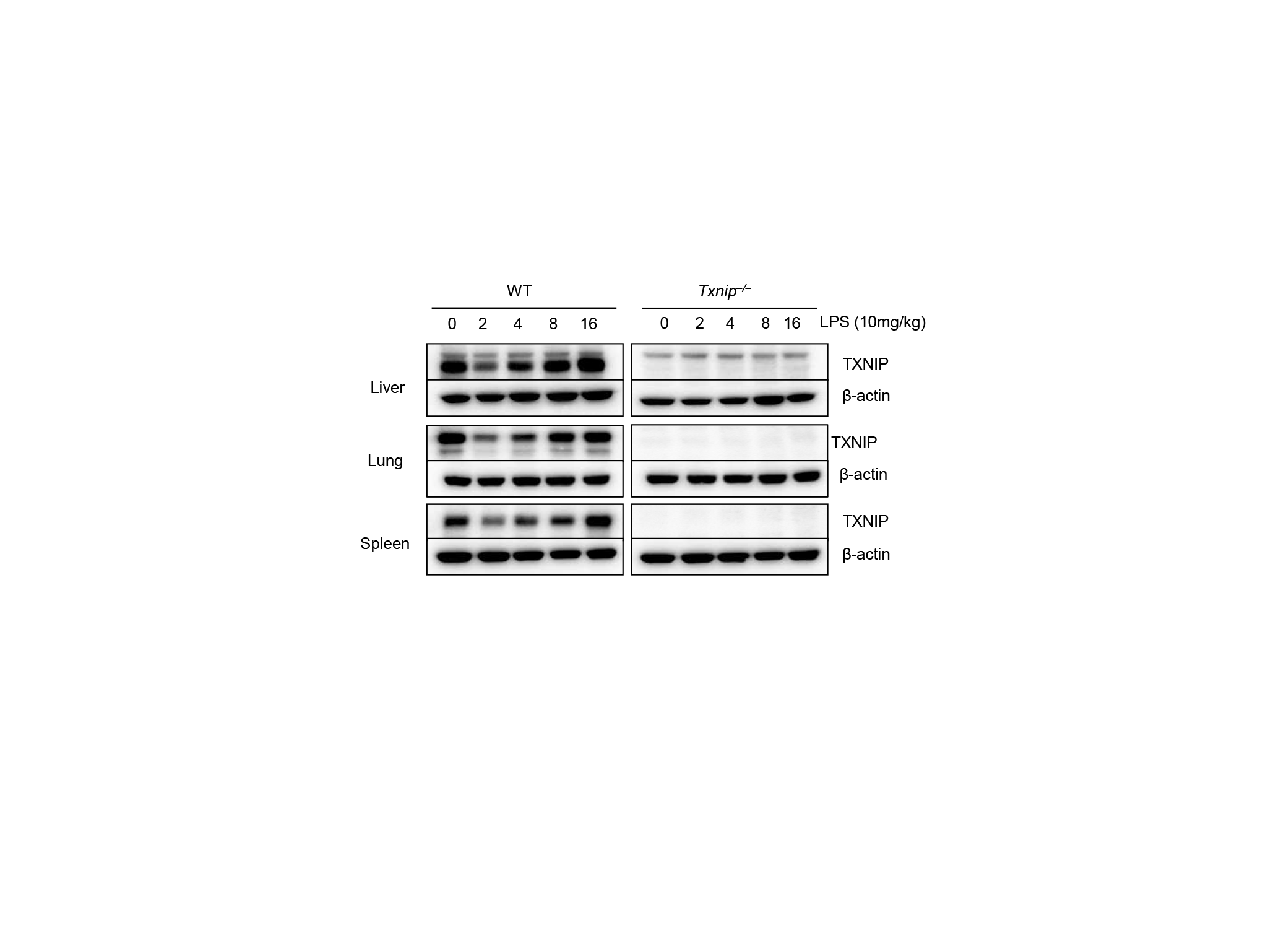

Supplement: Figure S1 — The expression levels of TXNIP after LPS administration in vivo . Liver, lung, and spleen samples were obtained from WT and Txnip−/− mice at 0, 2, 4, 8, and 16 h after LPS administration. Tissue lysates were prepared by homogenizing treatment and were then analyzed by immunoblotting with an anti-TXNIP antibody. These data are representative of at least 3 independent experiments. (TIF) [file ppat.1003646.s001.tif]

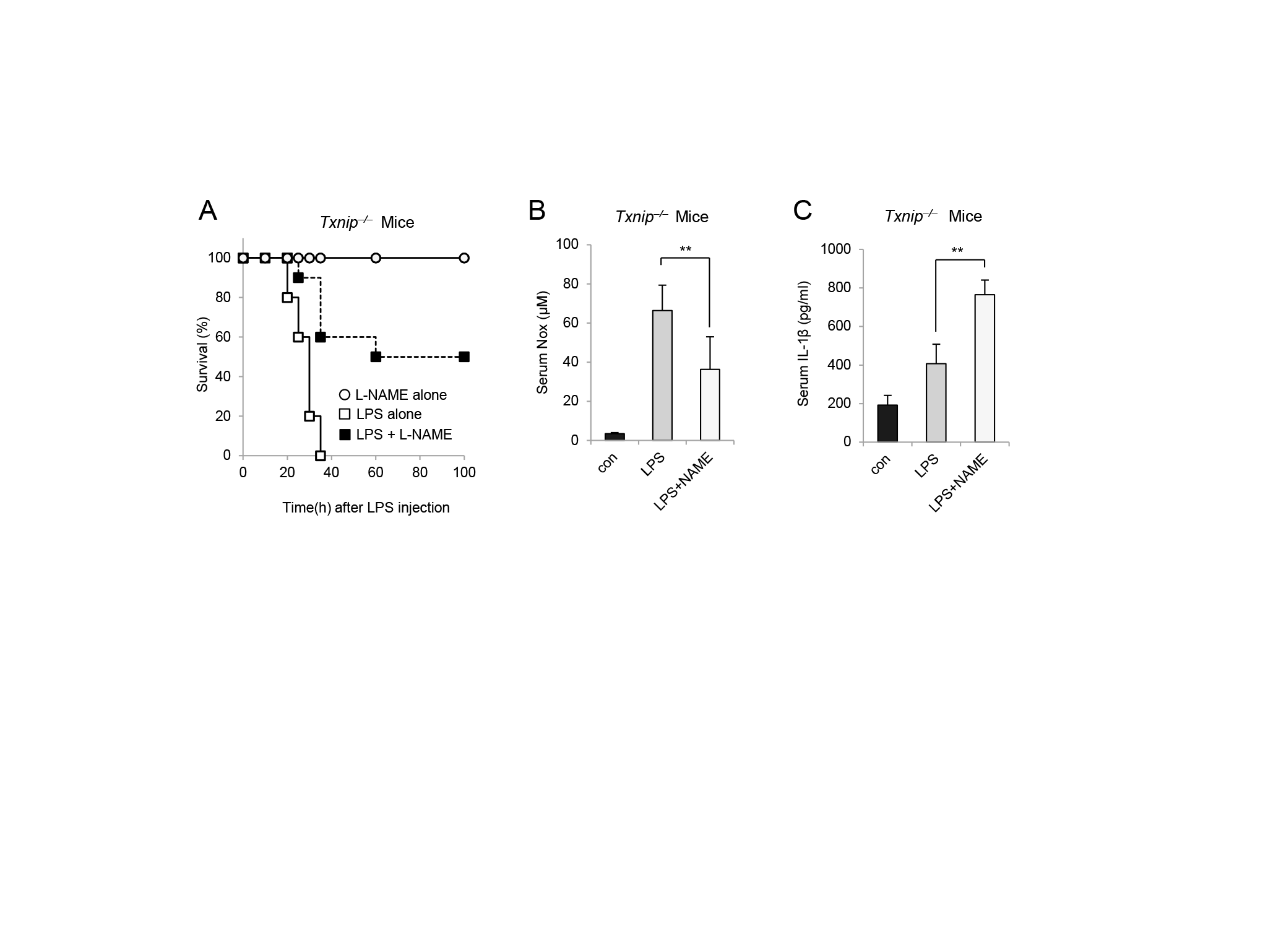

Supplement: Figure S2 — An iNOS inhibitor (L-NAME) rescues Txnip−/− mice injected with LPS in vivo . (A) L-NAME (30 mg/kg body weight) treatment was performed 1 h prior to LPS (10 mg/kg body weight; i.p.) injection (L-NAME group, n = 7 and LPS-alone and LPS+L-NAME group, n = 10). Animal viability was assessed every 5 h. The concentrations of serum NO (B) and IL-1β (C) in Txnip−/− mice (n = 5 per group) were determined. After LPS injection, the serum concentrations of NO and IL-1β were significantly altered by L-NAME treatment. These data are representative of at least 3 independent experiments (**P<0.01). (TIF) [file ppat.1003646.s002.tif]

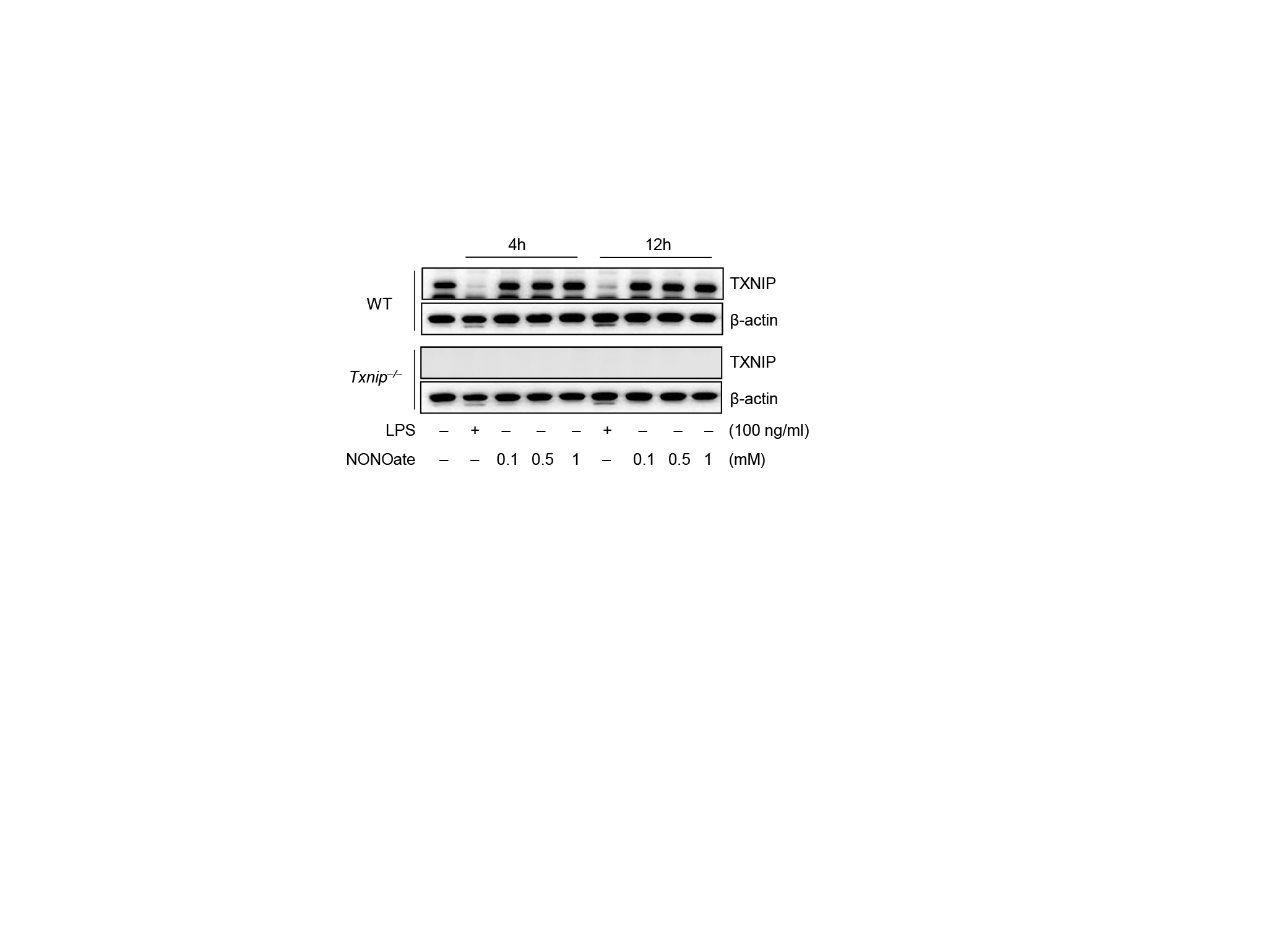

Supplement: Figure S3 — TXNIP does not affect the production of NO by the NO donor DETA-NONOate. Macrophages from WT or Txnip−/− mice were incubated with the NO donor DETA-NONOate at the indicated concentrations. The expression of TXNIP was measured by western blot with an TXNIP antibody. These data are representative of at least 3 independent experiments. (TIF) [file ppat.1003646.s003.tif]

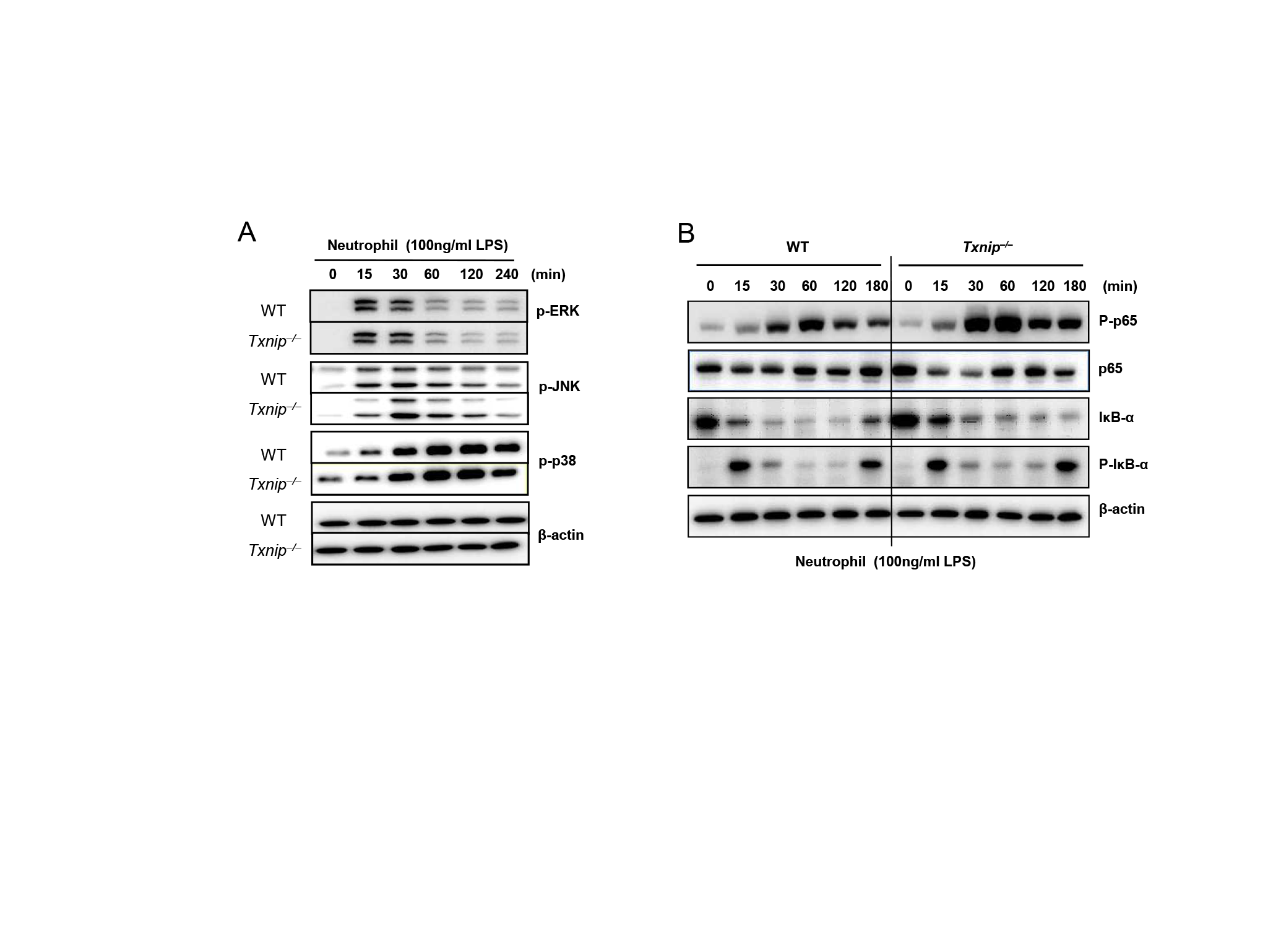

Supplement: Figure S4 — Signaling molecules activated in neutrophils after LPS treatment. BM neutrophils from WT or Txnip−/− mice were treated with 100 ng/ml LPS, and cell lysates were harvested at the indicated time points. (A) The levels of phosphorylated ERK, JNK, and p38 were determined by western blotting. (B) Western blot analysis was performed using anti-p65 and anti-IκBα antibodies. The detection of β-actin in each sample served as a loading control. Data are representative of at least 3 independent experiments. (TIF) [file ppat.1003646.s004.tif]

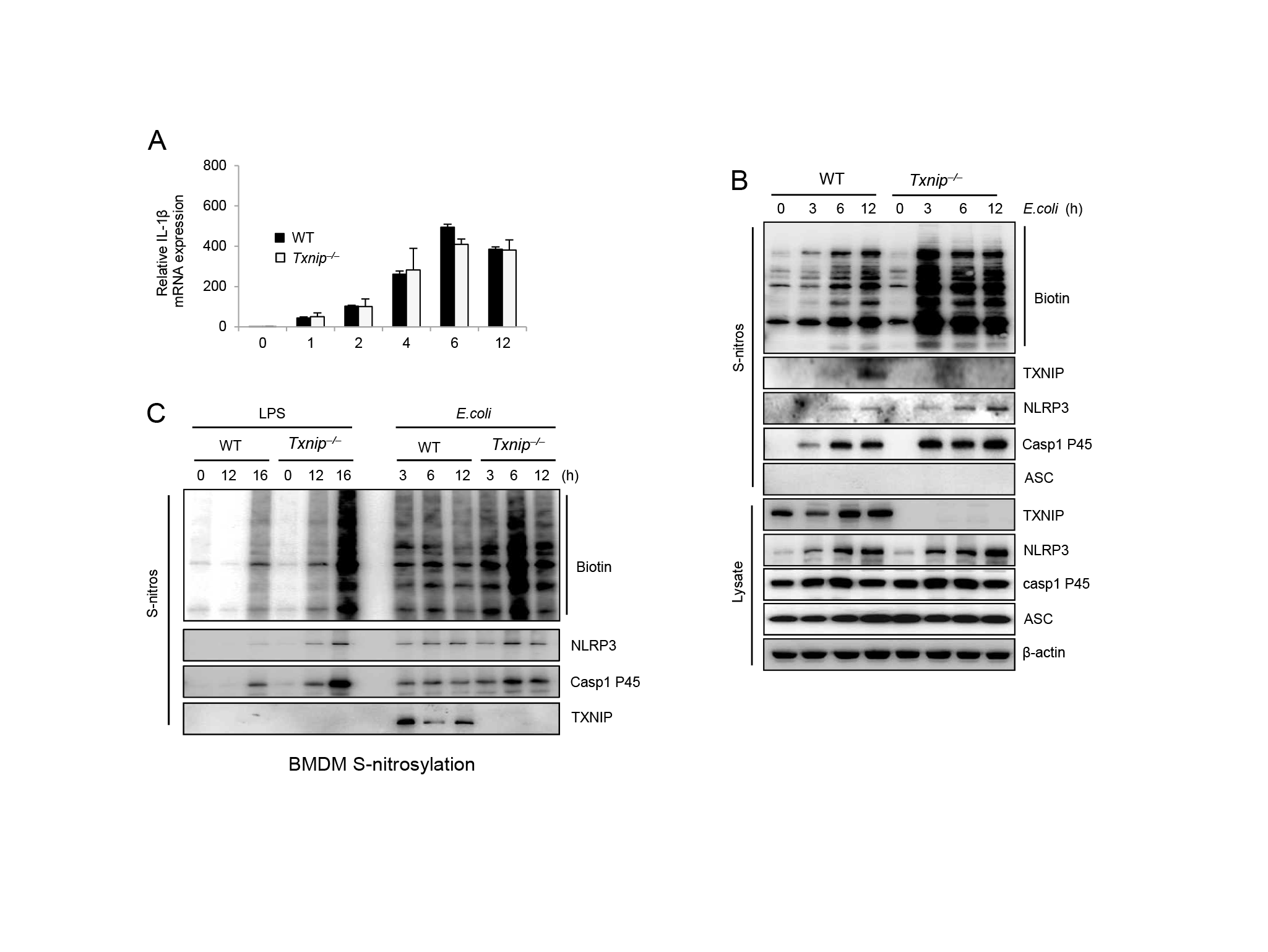

Supplement: Figure S5 — S-nitrosylation of inflammasome components on NO. (A) IL-1β mRNA levels of macrophages were determined by real-time PCR analysis. Peritoneal macrophages from WT or Txnip−/− mice were incubated with LPS (100 ng/ml) for the indicated time periods. (B) The total level of S-nitrosylation of inflammasome components was determined by immunoblot analysis. E. coli was used to treat peritoneal macrophages from WT and Txnip−/− mice at the indicated time points. Below (lysate), immunoblot analysis of total lysate fractions. Data are representative of at least 3 repeated experiments. (C) Total S-nitrosylation of inflammasome components, including NLRP3, caspase-1, and TXNIP was determined by immunoblot analysis. LPS or E. coli (MOI 10) was used to treat BMDMs from WT and Txnip−/− for the indicated time periods. Data are representative of at least 3 repeated experiments. (TIF) [file ppat.1003646.s005.tif]

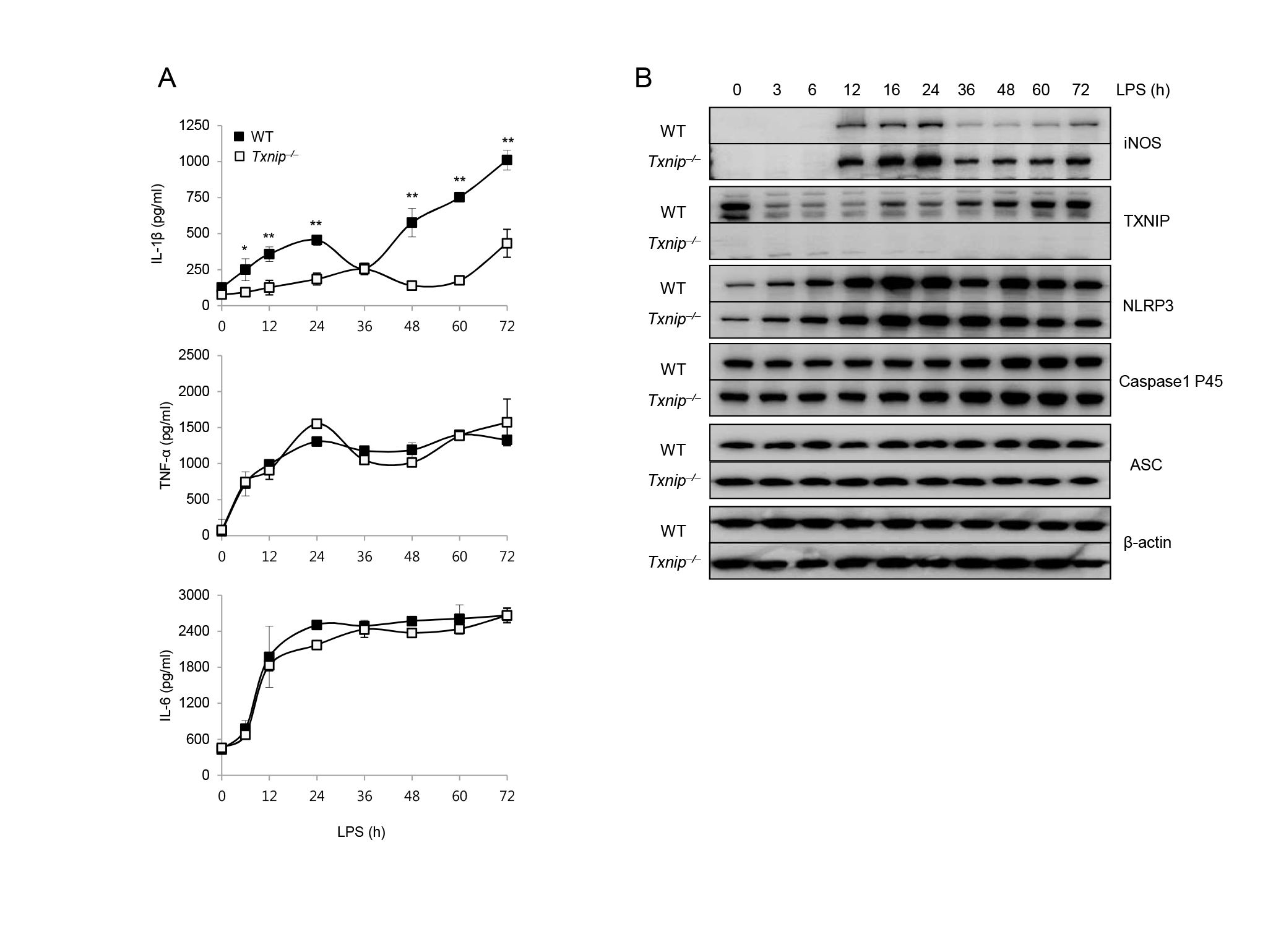

Supplement: Figure S6 — TXNIP affects the production of IL-1β and iNOS following LPS treatment in macrophages. Peritoneal macrophages from WT or Txnip−/− mice were treated with 100 ng/ml LPS, and the supernatants and cell lysates were harvested at the indicated time points (long-term). (A) The levels of IL-1β, TNF-α, and IL-6 in the culture supernatants were measured by ELISA. (B) Immunoblot analysis of iNOS, TXNIP, NLRP3, caspase-1, and ASC protein expression in macrophages. β-actin served as the loading control. Data are presented as the mean ± SD of 3 independent experiments (*P<0.05; **P<0.01) (TIF) [file ppat.1003646.s006.tif]
